# AI Quantification of Vascular Lesions in Mouse Fundus Fluorescein Angiography

**DOI:** 10.1167/tvst.14.6.4

**Published:** 2025-06-02

**Authors:** Vinodhini Jayananthan, Tyler Heisler Taylor, David Henry Greentree, Bryce Collison, Nagaraj Kerur

**Affiliations:** 1 Department of Ophthalmology and Visual Sciences, College of Medicine, The Ohio State University Wexner Medical Center, Columbus, OH, USA; 2 Department of Neuroscience, College of Medicine, The Ohio State University Wexner Medical Center, Columbus, OH, USA; 3 Department of Microbial Infection and Immunity, College of Medicine, The Ohio State University Wexner Medical Center, Columbus, OH, USA

**Keywords:** age-related macular degeneration, fluorescein angiography, AI

## Abstract

**Purpose:**

Quantifying vascular leakage in fundus fluorescein angiography (FFA) is a critical endpoint in preclinical models of diseases such as neovascular age-related macular degeneration, retinopathy of prematurity, and diabetic retinopathy. Traditional manual methods are labor intensive and prone to variability. We developed an artificial intelligence (AI)-assisted method to improve efficiency and accuracy in quantifying vascular lesions in FFA images.

**Methods:**

Nikon NIS-Elements software with AI functionality was used to create an automated FFA analysis method. FFA images were acquired using the Phoenix MICRON IV imaging system in two mouse models of ocular angiogenesis: (1) very low-density lipoprotein receptor (*Vldlr*) knockout mice exhibiting spontaneous pathological chorioretinal neovascularization, and (2) a laser-induced choroidal neovascularization model. The AI model was trained on manually segmented FFA images to delineate lesions and quantify lesion area and fluorescence intensity.

**Results:**

The AI model demonstrated high accuracy in quantifying vascular lesions in FFA images, achieving 99.7% agreement with manual counts. It attained a precision, recall, and F1 score of 0.94, with an intraclass correlation coefficient (ICC) of 0.991. The model showed strong spatial agreement with manual segmentations and consistent lesion area measurements. On validation images, it maintained expert-level performance (ICC = 0.998) with high sensitivity and precision. Additionally, it effectively captured temporal changes in vascular leakage by measuring lesion area and fluorescence intensity, demonstrating robustness in real-world experiments.

**Conclusions:**

Our AI model quantifies vascular lesions in FFA images with high accuracy, outperforming manual analysis.

**Translational Relevance:**

AI-based quantification provides a scalable, consistent alternative to manual methods, enhancing research efficiency.

## Introduction

Abnormal growth and leakage of blood vessels are characteristic features of several sight-threatening ocular conditions, including neovascular age-related macular degeneration (nvAMD), retinopathy of prematurity, and diabetic retinopathy.^1^ Fundus fluorescein angiography (FFA) highlights the retinal blood vessels, allowing for detection of abnormal growth and leakage. The traditional methods for quantifying vascular lesions involve manual delineation and measurement of the lesional area in each image. This approach of lesion quantification in FFA images is not only labor intensive and time consuming but also prone to variability and user bias. Additionally, this manual approach limits the feasibility of large-scale studies and introduces inconsistencies.

Recent advancements in artificial intelligence (AI) and machine learning have opened new possibilities for automating image analysis. AI-based tools have shown impressive performance in analyzing and quantifying pathological features across various imaging modalities, including radiology, pathology, and ophthalmology.^2^ For FFA assessment in preclinical animal models, AI-based workflows can potentially overcome limitations associated with manual quantification, providing a faster, repeatable, and standardized approach to analyzing vascular lesions.

In this study, we developed an AI-based analysis method designed for the automated quantification of vascular lesions in mouse FFA images. Specifically, we focused on two preclinical mouse models relevant to nvAMD: the very low-density lipoprotein receptor knockout (*Vldlr^−/−^*) mouse, and a laser-induced choroidal neovascularization (CNV) model.^3^^–^^5^ Although the neovascularization in the laser CNV model mimics the pathology of type 2 nvAMD, where vessels penetrate the retinal pigment epithelium (RPE) and leak into the subretinal space, the lesions in the *Vldlr^−/−^* model resemble type 3 nvAMD, characterized by neovascularization originating from the deep retinal capillary plexus and extending downward toward the RPE. Our AI-based method accurately detects and quantifies vascular abnormalities in these models, allowing automated analysis of large number of images.

## Methods

### Animals

Male and female C57BL/6J wild-type (WT) mice 8 to 12 weeks old and male and female *Vldlr^−/−^* mice 3 to 11 weeks old were used in this study. Both C57BL/6J and *Vldlr^−/−^* mice were obtained from The Jackson Laboratory (Bar Harbor, ME). All experimental procedures were approved by the Institutional Animal Care and Use Committee of The Ohio State University. Studies were conducted in accordance with the principles outlined in the ARVO Statement for the Use of Animals in Ophthalmic and Vision Research. The mice were housed in a standard sterile facility with ventilated cage systems. The environment maintained a controlled temperature and humidity, with a 12-hour light/12-hour dark cycle. The animals had access to sterilized, irradiated rodent diet (a standard laboratory mouse diet) ad libitum and received sterile water through automatic water systems.

### Laser-Induced Mouse Model of Choroidal Neovascularization

Laser photocoagulation using the MICRON IV Image-Guided Laser System (Phoenix-Micron, Bend, OR) was performed following a previously established procedure.^6^ This system utilizes a Merilas 532 green laser photocoagulator (Meridian Medical, Thun, Switzerland). Laser photocoagulation lesions were induced with spot diameter of 50 µm, delivering a 70-ms pulse of 220-mW power. Prior to each experiment, the laser system was calibrated to ensure accurate power output. The mice were anesthetizedwith Avertin (500 µg/g body weight) via intraperitoneal injection. Their eyes were then dilated with tropicamide and phenylephrine drops, and a general eye lubricant (GenTeal Tears) was applied to the cornea. When the pupils were dilated, the mice were positioned to visualize fundus on a computer screen connected to the MICRON IV system, and the laser beam was directed onto the fundus. Four laser burns were created around the optic disc at the 3, 6, 9, and 12 o'clock positions, approximately 2 to 3 optic disc diameters away from the optic nerve. Confirmation of the Bruch's membrane rupture was observed by the formation of a bubble at the center of the laser spot. Following the laser procedure, mice were placed under a heat lamp until they recovered from anesthesia and were returned to the vivarium.

### *Vldlr^−/−^* Mouse Model of Spontaneous Retinal Neovascularization

In *Vldlr^−/−^* mice, abnormal blood vessels from the deep retinal vasculature within the outer plexiform layer continue to proliferate, forming choroidal anastomosis typically by 2 months of age, mimicking the features of nvAMD and retinal angiomatous proliferation.^4^^,^^7^^,^^8^

### FFA Imaging

FFA images were captured from both the *Vldlr^−/−^* and laser-induced CNV mouse models using the MICRON IV imaging system. For the laser-induced CNV model, FFA images were taken on day 3 or day 7 post-laser. For the *Vldlr^−/−^* mice, images were taken at 3 to 11 weeks of age . Mice were anesthetized and pupils were dilated with topical drops of tropicamide and phenylephrine as described above. Mice were then intraperitoneally administered with fluorescein (30 µg/gm of body weight, Fluorescite 10%), and FFA images were captured at 5 and 9 minutes for the right eye, and at 6 and 10 minutes for the left eye following fluorescein injection. To ensure a diverse dataset, images used for AI training and validation were acquired with a gain setting range of 0 to 3 and frame rate of 30 fps. For temporal leakage analysis, images were consistently captured at a fixed gain setting of 0 and a frame rate of 30 fps.

### Image Calibration

All FFA images used in this study were captured using the MICRON IV fundus camera. The original TIFF files were imported into the NIS-Elements software (Nikon, Tokyo, Japan) and subsequently converted to ND2 format for further analysis. Image calibration was performed within the software, using a pixel size of 2.1 µm per pixel to ensure accurate spatial measurements during downstream processing.

### Training Dataset and Manual Segmentation

FFA images from laser CNV in WT and *Vldrl^−/−^* mouse models were manually segmented to delineate vascular lesions using the NIS-Elements manual segmentation tool. The NIS.ai suite within the NIS-Elements software was trained on a dataset of manually segmented FFA images identifying vascular lesions, which served as the ground truth. To account for interoperator variability, seven of these images were manually segmented independently by three different operators. Each of these operator-marked segmentations was treated as a unique training image, resulting in 21 images for training from these seven original images. Additionally, two other images were manually segmented independently by two operators, yielding four images for training from this subset. Finally, 55 unique images, each manually segmented by a single operator, were included in the training dataset. Overall, this segmentation strategy resulted in a final training set of 70 images, incorporating interoperator variability into the ground truth to enhance the ability of the model to generalize. Objects smaller than 60 µm^2^ were not included to eliminate artifacts. Across these 70 images, 1796 vascular lesions were identified and manually segmented. In each FFA image, the number of vascular lesions, lesion area, and mean fluorescence intensity were computed.

### Images for Validation

Nine images not included in the training set were manually segmented by a single operator to validate the AI method. These images contained a total of 127 FFA lesions, all manually annotated by the same operator.

### Performance Evaluation Metrics

A comprehensive set of metrics was employed to evaluate the performance of the AI method against the manual approach. These metrics included (1) metrics of sensitivity and specificity, capturing the ability of the model to correctly identify true positive and true negative pixels; (2) metrics of spatial agreement between manual and AI methods; and (3) metrics of lesion area measurement accuracy.

### Metrics of Lesion Detection Accuracy and Sensitivity

The object (i.e., vascular lesion) prediction accuracy of the model was assessed using precision, recall, and F1 score metrics. Precision measures the proportion of correctly predicted lesions out of all predicted lesions, and recall measures the proportion of correctly predicted lesions out of all actual lesions. Both metrics are based on the concept of true positives (TPs), false positives (FPs), and false negatives (FNs). To balance both precision and recall, we also calculated the F1 score, a harmonic mean of precision and recall.

#### Precision

Precision measures the accuracy of the AI method in predicting the presence of an object (i.e., vascular lesion in this context) in the image. It is the ratio of number of correctly predicted objects (TPs) to all objects predicted by the AI (TP + FP):

$$\begin{array}{c} \mathrm{Precision}=\frac{\mathrm{TP}}{\mathrm{TP}+\mathrm{FP}} \end{array}$$

The precision value ranges from 0 to 1, where

• If precision = 1, there were no FPs (AI correctly identified all objects without extra detections).
• If precision = 0, none of the predicted positives was correct (all FPs).

#### Recall (Sensitivity)

Recall measures the ability of the AI method to correctly identify true positives. It is the ratio of correctly predicted objects (TPs) to all actual objects (TP + FN):

$$\begin{array}{c} \mathrm{Recall}=\frac{\mathrm{TP}}{\mathrm{TP}+\mathrm{FN}} \end{array}$$

The recall value ranges from 0 to 1, where

• If recall = 1, all actual positives were correctly predicted (no FNs).
• If recall = 0, none of the actual positives was predicted (all FNs).

#### F1 Score

A harmonic mean of precision and recall, the F1 score provides a single metric that balances both precision and recall, giving an overall assessment of the performance of the model.

$$\begin{array}{c} F1\;\mathrm{Score}=2\;\times \;\frac{\mathrm{Precision}\;\times \;\mathrm{Recall}}{\mathrm{Precision}+\mathrm{Recall}} \end{array}$$

The F1 score ranges from 0 to 1, where

• F1 = 1 indicates perfect precision and recall, meaning the model made no FPs or FNs (all positive predictions were correct and all actual positives were detected).
• F1 = 0 indicates that either precision or recall was 0, meaning either the model had no correct positive predictions (precision = 0) or it failed to detect any TPs (recall = 0).

### Metric of Spatial Agreement Between Manual and AI Methods

To quantitatively assess the spatial overlap between manual and AI-predicted segmentations, we calculated the intersection over union (IoU). IoU is a precise metric for spatial agreement through measuring the ratio of the area of overlap between the two segmentations to their combined area:

$$\begin{array}{c} \mathrm{IoU}=\frac{\mathrm{Area}\;\mathrm{of}\;\mathrm{intersection}}{\begin{array}{c} \mathrm{Area}\;\mathrm{of}\;\mathrm{manual}\;\mathrm{segmentation}\\ \;+\;\mathrm{Area}\;\mathrm{of}\;\mathrm{AI}\;\mathrm{prediction}-\mathrm{Area}\;\mathrm{of}\;\mathrm{intersection} \end{array}} \end{array}$$

The IoU value ranges from 0 to 1, wheres

• If IoU = 1, the segmentations are identical.
• If IoU = 0, there is no overlap between the two segmentations.

### Metrics of Quantitative Difference in the Lesion Area Measurement Between Manual and AI Methods

The differences in lesion area measurements between the manual and AI-based segmentation methods were quantified using percentage error (PE). PE expresses the discrepancy between the ground truth and AI method lesion areas as a percentage of the ground truth, offering a clear and standardized measure of the magnitude of deviation between AI predictions and manual segmentations:

$$\begin{array}{c} \mathrm{PE}=\frac{\mathrm{GT}\;\mathrm{area}-\mathrm{AI}\;\mathrm{area}}{\mathrm{GT}\;\mathrm{area}}\times \;100 \end{array}$$

where GT area is the ground truth area of the lesion, and AI area is the AI-predicted area of the lesion.

### Measurement of Leakage Intensity

Vascular leakage intensity in FFA lesions was quantified using pixel-based mean fluorescence intensity (MFI). Regions of interest (ROIs), delineating individual lesion boundaries, were established through manual or AI-based segmentation. The MFI was then calculated for each ROI, providing a measure of leakage intensity per lesion, as well as an average MFI across all lesions within each image.

$$\begin{array}{c} \mathrm{MFI}=\;\frac{\Sigma \left(\mathrm{Pixel}\;\mathrm{intensities}\right)}{\mathrm{Number}\;\mathrm{of}\;\mathrm{pixels}} \end{array}$$

### Intraclass Correlation Coefficient

To evaluate the agreement between AI-predicted ground truth and manual measurements, the intraclass correlation coefficient (ICC) was computed for both lesion prediction and lesion area measurement. The ICC is a statistical measure that assesses both the consistency and absolute agreement between the two methods, providing insight into how well the AI-predicted lesion count and areas align with the manual method. Together these metrics offer a comprehensive evaluation of the accuracy of the AI model in area prediction and the reliability of its performance compared to the ground truth. An ICC of 1 indicates perfect agreement (identical counts for all images), whereas an ICC of 0 signifies no agreement beyond random chance.

### Instructions for Implementing the AI-Based Analysis Method

To facilitate the seamless implementation of our AI-based analysis, we provide the following resources and instructions: (1) Segment Objects AI (.oai) file, which contains the trained AI model optimized for delineating FFA lesions Supplementary Files AI model 2.0.zip; (2) General Analysis 3 (.ga3) file, which defines the analysis workflow, including automated lesion counting, area measurement, intensity analysis, and data tabulation Supplementary Files AI Analysis FFA Lesion area X Intensity with CSV export FINAL.zip and fundus calibration.zip; and (3) a step-by-step guide that provides detailed instructions covering software setup, image preparation, analysis execution, and result tabulation.

## Results

### AI Performance in Lesion Detection in Training Images

Seventy mouse FFA images containing 1796 manually demarcated vascular lesions were used to train the segmentation model via the Train Segment Objects.ai feature in the NIS-Elements software. To ensure accurate AI-predicted lesions, an AI-predicted lesion was considered true positive only if it overlapped with at least half of the combined area delineated by both manual and AI methods. This overlap was measured using an IoU threshold of 0.5.

**Figure 1.**
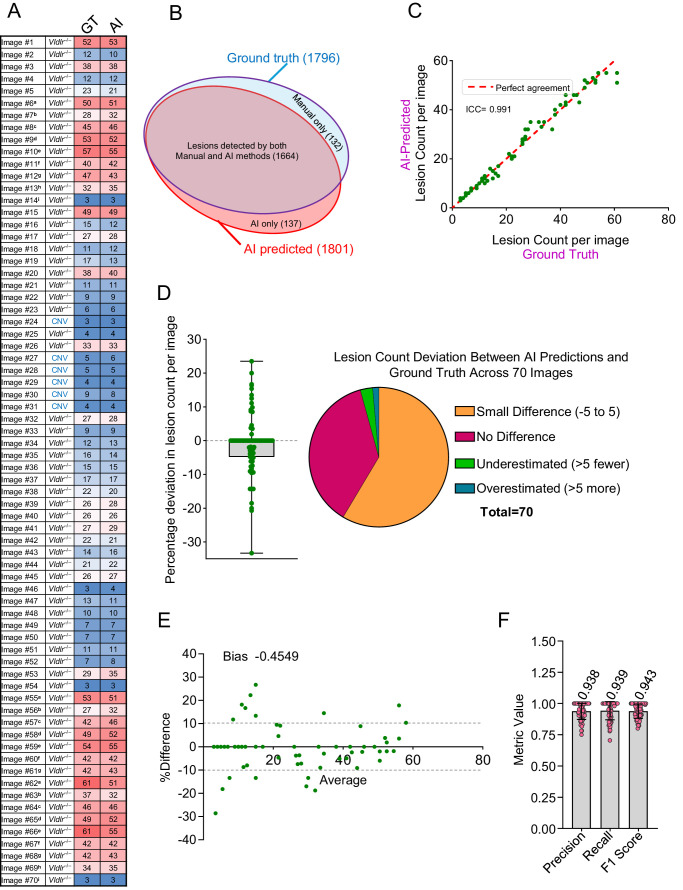
Evaluation of the AI method performance in lesion detection on training images. (**A**) Heatmap showing the lesion count in each of the 70 training images. (**B**) Venn diagram illustrating lesion detection overlap between AI and manual methods. (**C**) Correlation analysis of lesion counts per image between the ground truth and AI methods, quantified by the ICC. (**D**) Distribution of percentage deviation in AI-predicted lesion counts per image compared to the ground truth, represented using a box-and-whisker plot and a pie chart. (**E**) Bland–Altman plot comparing lesion counts per image between the ground truth and AI methods. (**F**) Performance metrics, including precision, recall, and F1 score, for lesion detection by the AI method.

Performance analysis demonstrated a strong agreement between AI predictions and ground truth lesion counts. The AI method detected 1801 lesions across 70 training images compared to ground truth, which identified 1796 lesions, indicating 99.7% agreement in lesion count overall (Fig. 1A). Of the 1801 AI-predicted lesions, 137 lesions were not identified by the manual method (Fig. 1B). Furthermore, AI failed to detect 132 of the manually marked lesions (Fig. 1B). A detailed comparison of lesion counts across all 70 images demonstrated excellent agreement, with an ICC of 0.991, calculated using a two-way mixed-effects model for absolute agreement (Fig. 1C).

**Table.** Performance Metrics for the AI Method, Including Results on Training and Validation Images and Interoperator Comparisons

	Manual vs. AI Comparison		Interoperator Comparison		
	Training Dataset	Validation Dataset	User #1 vs. User #2	User #3 vs. User #1	User #3 vs. User #2
Lesion count agreement (ICC)	0.991	0.998	0.966	0.777	0.752
Lesion count per image bias (Bland–Altman analysis)	−0.45	−5.62	3.24	6.65	9.89
Lesion prediction precision	0.938	0.90	0.82	0.82	0.80
Lesion prediction recall	0.939	0.96	0.79	0.77	0.73
Lesion prediction F1 score	0.943	0.93	0.81	0.79	0.76
Lesion area per image agreement (ICC)	0.994	0.990	0.880	0.964	0.937
Individual lesion area agreement (ICC)	0.988	0.976	0.880	0.927	0.896
Lesion area measurement per image bias (Bland–Altman analysis)	0.09	−4.22	13.3	−0.91	11.58
Individual lesion area measurement bias (Bland–Altman analysis)	0.47	6.3	9.65	−2.04	5.65
Spatial agreement at lesion area per image (mean IoU)	0.89	0.76	0.69	0.70	0.68
Spatial agreement at individual lesion area (mean IoU)	0.86	0.73	0.69	0.70	0.70

Analysis at the individual image level revealed that exact matches in object counts were observed in 37.1% of the images (26 out of 70). Additionally, 58.6% of the images (41 out of 70) showed a minor discrepancy (±5 lesions), resulting in over 95% of images demonstrating minimal differences. In contrast, two images (2.9%) were classified as underestimated by the AI, with more than five fewer lesions predicted compared to the ground truth. Conversely, one image (1.4%) was classified as overestimated, with the AI predicting more than five additional lesions compared to the ground truth (Fig. 1D). The Bland–Altman analysis revealed that the AI method slightly underestimated lesion counts compared to the manual method, with a mean bias of −0.45% (Fig. 1E).

Further examination of images with count discrepancies revealed that these differences largely stemmed from the merging of closely located lesions, where accurately defining boundaries posed challenges. Our training dataset included several images independently segmented by three operators to account for interoperator variability. These images, which included merged lesions, also displayed variation in lesion counts across operators, suggesting that separating some merged lesions into distinct entities is inherently subjective (Supplementary Figs. S1, S2). Notably, despite count variations among the three human operators, AI-predicted lesion counts remained consistent, even when the same image was presented multiple times (each instance corresponding to a different operator's annotation) in the training dataset (Supplementary Fig. S1). This consistency underscores the robustness of the AI model in lesion counting, demonstrating that it provides a deterministic, repeatable method of quantification, ensuring reliable results even in the presence of complex lesion morphologies and interoperator differences.

The lesion detection performance of the AI model was further evaluated using key accuracy and sensitivity metrics: precision, recall, and F1 score. Across the 70 analyzed images, the model achieved an average precision of 0.94, a recall (sensitivity) of 0.94, and an F1 score of 0.94 (Fig. 1F), underscoring its high accuracy and consistency in accurately identifying vascular lesions. A precision of 0.94 indicates that, when the model predicts a lesion, it is correct 94% of the time. Similarly, a recall of 0.94 means the model successfully detects 94% of all actual lesions. The high F1 score, as a harmonic mean of precision and recall, confirms the balanced performance of the model in both identifying true lesions and avoiding incorrect detections. These metrics demonstrate the robustness and reliability of the model in detecting lesions with minimal FPs and missed detections.

### Quantifying Spatial Agreement Between Manual and AI-Predicted Lesions With IoU

The spatial agreement between manual and AI-predicted lesion segmentations was assessed using IoU. IoU was selected over the dice similarity coefficient because of its more stringent requirements for spatial overlap and its extensive use in the literature for evaluating segmentation accuracy in object boundaries in medical imaging.^9^^–^^13^ To account for potential variations in lesion delineation approaches (example lesions are identified in Supplementary Fig. S2), such as cases where one method merged multiple lesions into a single region but the other separated them, an adapted IoU calculation was applied. For instances of merged lesions, the aggregated area of individual lesions nested within the bigger marking was used to compare with the merged region. This approach allowed for meaningful evaluation of spatial agreement between manual and AI-derived lesion boundaries, even when segmentation styles differed.

**Figure 2.**
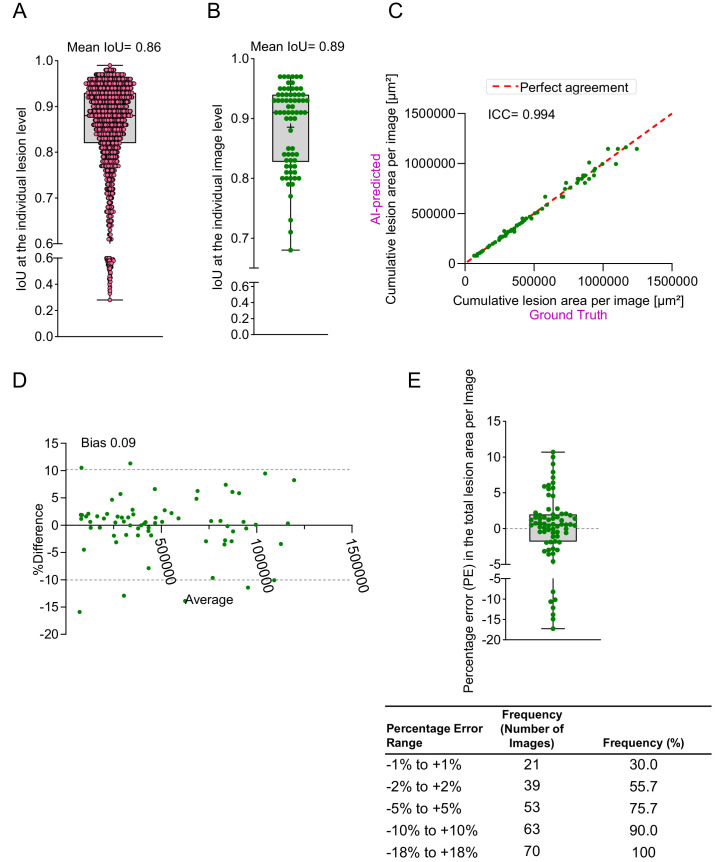
Evaluation of the AI method performance in lesion spatial agreement and area on training images. (**A**) Spatial agreement at the level of individual lesion between ground truth and AI method quantified by IoU. (**B**) Spatial agreement at the level of individual image between ground truth and AI method quantified by IoU. (**C**) Correlation analysis of lesion area per image between the ground truth and AI methods, quantified by the ICC. (**D**) Bland–Altman plot comparing lesion area per image between the ground truth and AI methods. (**E**) Distribution of percentage error in AI-predicted lesion area per image compared to the ground truth, represented using a box-and-whisker plot and frequency table.

As described above, the AI segmentation model was trained on 70 images containing 1796 manually demarcated lesions. At the individual lesion level, AI predictions demonstrated high fidelity, with an average IoU of 0.86 (95% confidence interval [CI], 0.85–0.86). Notably, 80% of the observations recorded an IoU of 0.8 or higher, highlighting the strong spatial agreement of the model with manual annotations (Fig. 2A). At the individual image level, the overall spatial agreement of the lesion areas further improved, achieving an average IoU of 0.89 (95% CI, 0.87–0.90), suggesting a strong agreement in lesion boundaries across entire FFA images (Fig. 2B).

### Quantitative Assessment of Lesion Area Measurement: AI Versus Manual Methods

The AI segmentation model, as described above, demonstrated high precision in detecting vascular lesions, achieving an F1 score of 0.94 (Fig. 1F) and a strong spatial agreement with manual segmentations, reflected by an IoU of 0.86 (Figs. 2A, 2B). By determining ICC, bias (Bland–Altman analysis), and percentage error, we have quantitatively assessed the accuracy of lesion area measurements by the AI model. This was performed both at the overall image level (cumulative lesion area for each FFA image) and at the individual lesion level (individual lesions across all 70 images). PE represents the difference between the ground truth lesion area and the AI-estimated area, expressed as a percentage of the ground truth value. A positive PE value indicates that the AI underestimated the lesion area, and a negative PE shows overestimation. This metric helps to quickly assess the accuracy and reliability of the AI model relative to manual measurements across different lesion sizes.

### Image-Level Analysis (Cumulative Lesion Area Per Image)

Cumulative lesion area for each FFA image was calculated by summation of areas of individual lesions. Our AI-based method demonstrated an excellent agreement in the measurement of cumulative lesion area per image across all 70 training images, with an ICC of 0.994, calculated using a two-way mixed-effects model for absolute agreement (Fig. 2C). Next, a Bland–Altman analysis was performed to evaluate the agreement between the AI-based and manual methods for measuring cumulative lesion areas. The analysis revealed minimal bias of 0.09%, suggesting excellent overall agreement between the two methods (Fig. 2D). The 95% limits of agreement were calculated as −10.04% to 10.22%, meaning that, for most images, the AI method underestimated the lesion area by up to 10.04% or overestimated it by up to 10.22% compared to the manual method. The narrow range of the limits of agreement further supports the consistency of the AI measurements relative to the manual method.

We also assessed the deviation of the AI model from manual measurements by calculating PE. The model demonstrated strong accuracy, with 90% of images showing a deviation of no more than ±10% from the ground truth (Fig. 2E, Supplementary Fig. S3). Additionally, 55.7% of the images demonstrated a deviation within ±2%, emphasizing the reliability of the AI method for lesion area estimation (Fig. 2E, Supplementary Fig. S3).

### Lesion-Level Analysis (Individual Lesion Area)

**Figure 3.**
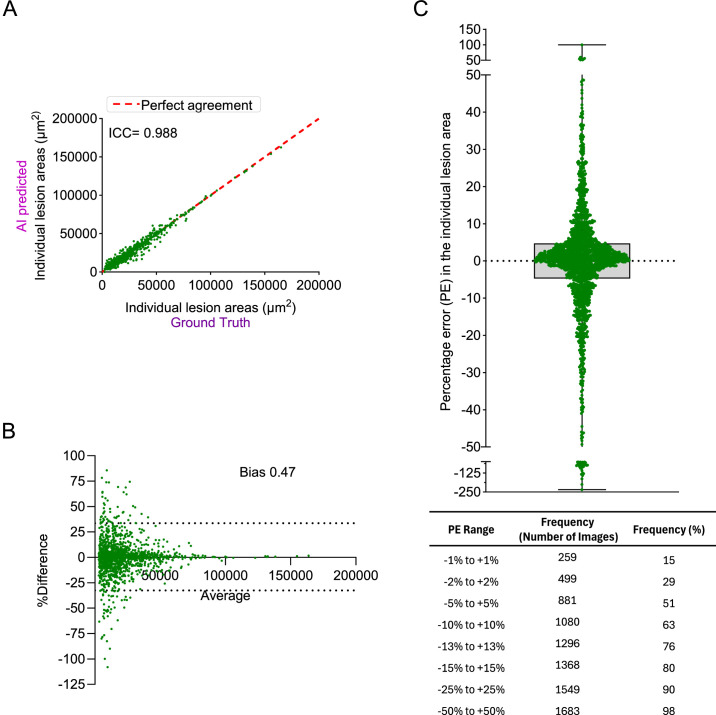
Evaluation of the AI method performance in individual lesion area measurement on training images. (**A**) Correlation analysis of individual lesion area measurement between the ground truth and AI methods, quantified by the ICC. (**B**) Bland–Altman plot comparing individual lesion area measurement between the ground truth and AI methods. (**C**) Distribution of percentage error in AI-predicted individual lesion area measurements compared to the ground truth, represented using a box-and-whisker plot and frequency table.

The AI method detected 1801 lesions across 70 images analyzed compared to ground truth, which identified 1796 lesions. In cases of merged lesions, the combined area of individual lesions nested within the bigger markings (example lesions identified in Supplementary Fig. S2) was used to compare with the merged region, ensuring accurate and fair comparison of lesion areas. The AI method demonstrated strong agreement in lesion area measurements, with an ICC of 0.988, calculated using a two-way mixed-effects model for absolute agreement (Fig. 3A). Bland–Altman analysis indicated a bias of 0.47%, with 95% limits of agreement ranging from −32.64% to 33.57% (Fig. 3B).

We examined PE across the 1664 lesions that were detected by both manual and AI methods (Fig. 3C). Nearly half of the lesions (51.4%) had predictions falling within a narrow deviation range of −5% to +5% relative to ground truth (negative values indicate over estimation, and positive values indicate underestimation) (Fig. 3C, Supplementary Fig. S4). Furthermore, 63% of lesions had predictions falling within a ±10% margin of error, and 80% of lesions had predictions within a ±15% range. Together, these findings reflect the consistency and reliability of the model in accurately estimating individual lesion areas (Fig. 3C, Supplementary Fig. S4A). For a broader ±25% range, 90.3% of lesion predictions remained within this boundary, highlighting robust accuracy across most cases, albeit with some variability. High-deviation outliers, where predictions deviated by more than +50%, represented a modest 2.3% of cases, indicating occasional overestimation or underestimation in lesion size (Fig. 3C, Supplementary Fig. S4A). Overall, the AI model performed with high accuracy at the image level. At the individual lesion level, however, variability was higher than at the image level, largely due to challenges in defining boundaries for closely clustered lesions.

### Comparative Evaluation of AI Performance on FFA Images From *Vldlr^−/−^* and Laser CNV Models

To assess potential differences in AI performance across the two models, we compared its ability to analyze FFA images from the *Vldlr^−/−^* and laser CNV models. The precision, recall, and F1 scores for both models were comparable, with the AI model performing slightly better on images from the laser CNV model (Supplementary Fig. S4B). Similarly, the IoU values for images from both models were comparable (Supplementary Figs. S4C, S4D), as were the PE value distribution ranges (Supplementary Figs. S4E, S4F). These results indicate that the performance of the AI model remained largely consistent across the *Vldlr^−/−^* and laser CNV models.

### AI Performance on Validation Images

**Figure 4.**
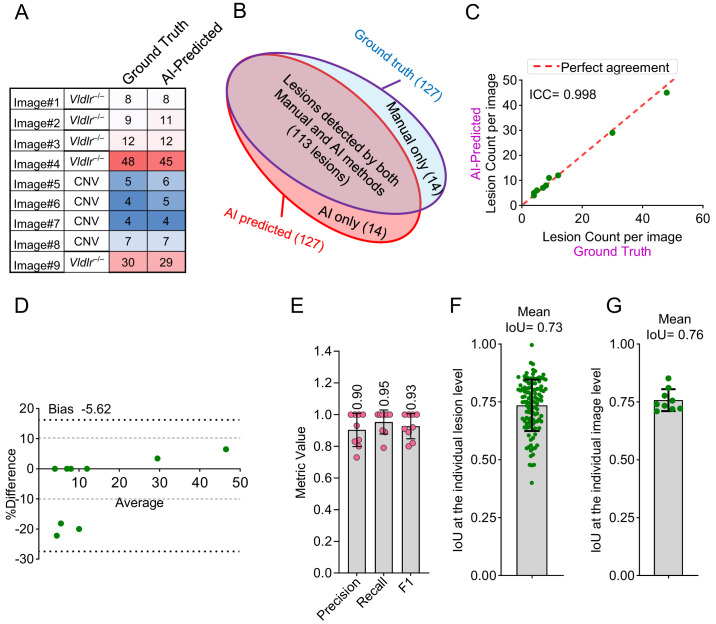
Evaluation of the AI method performance in lesion detection and spatial agreement on validation images. (**A**) Heatmap showing the lesion count in each of the nine validation images. (**B**) Venn diagram illustrating lesion detection overlap between AI and manual methods. (**C**) Correlation analysis of lesion counts per image between the ground truth and AI methods, quantified by the ICC. (**D**) Bland–Altman plot comparing lesion counts per image between the ground truth and AI methods. (**E**) Performance metrics, including precision, recall, and F1 score, for lesion detection by the AI method. (**F**) Spatial agreement at the level of individual lesion between ground truth and AI method quantified by IoU. (**G**) Spatial agreement at the level of individual image between ground truth and the AI method quantified by IoU.

Following the high accuracy observed during training, we next evaluated the performance of the AI model on a new set of validation images that were not included in the training dataset. The model was tested on nine validation images containing 127 manually annotated FFA lesions (Fig. 4A). The AI model identified 127 lesions, 113 of which corresponded to the manual annotations; however, the model missed 14 manually marked lesions and identified 14 additional lesions that were not part of the manual annotations (Fig. 4B).

**Figure 5.**
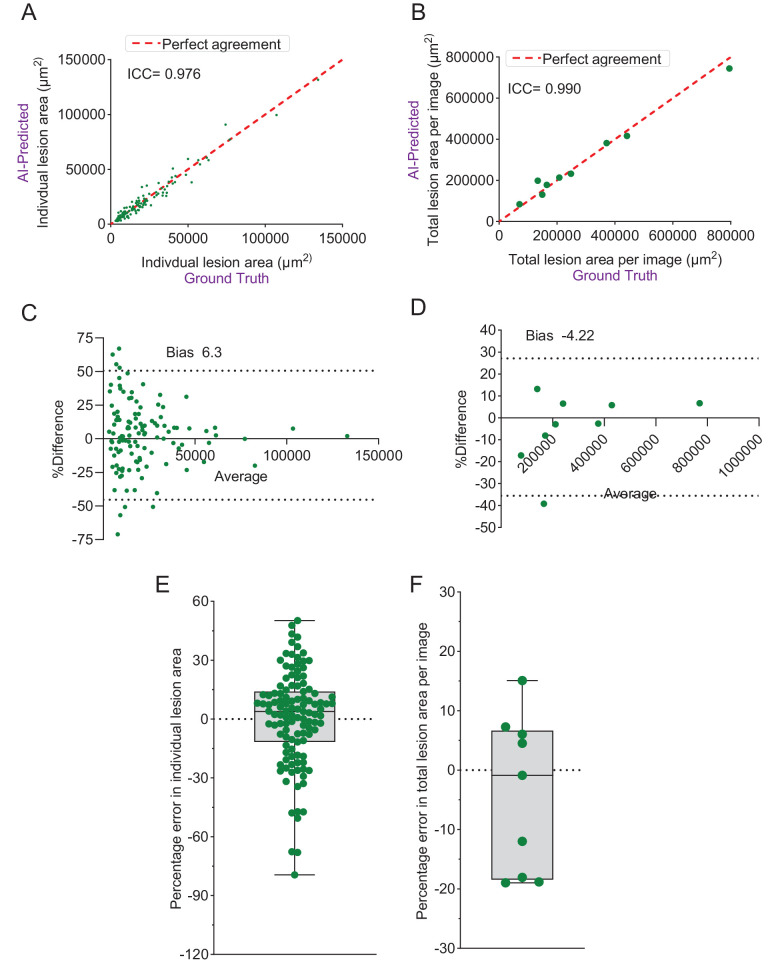
Evaluation of the AI method performance in lesion area measurement on validation images. (**A**) Correlation analysis of individual lesion area measurement between the ground truth and AI methods, quantified by the ICC. (**B**) Correlation analysis of image-wise cumulative lesion area measurement between the ground truth and AI methods, quantified by the ICC. (**C**) Bland–Altman plot comparing individual lesion area measurement between the ground truth and AI methods. (**D**) Bland–Altman plot comparing image-wise cumulative lesion area measurement between the ground truth and AI methods. (**E**) Distribution of percentage error in AI-predicted individual lesion area measurements compared to the ground truth, represented using a box-and-whisker plot. (**F**) Distribution of percentage error in AI-predicted image-wise cumulative lesion area measurements compared to the ground truth, represented using a box-and-whisker plot.

The AI model demonstrated strong agreement with manual lesion segmentation, achieving an ICC of 0.998 based on a two-way mixed-effects model for absolute agreement (Fig. 4C). Bland–Altman analysis revealed a bias of −5.62%, with 95% limits of agreement ranging from −27.46% to 16.22% (Fig. 4D). The model achieved an average precision, recall (sensitivity), and F1 scores of 0.90, 0.95, and 0.93, respectively, across the nine validation images (Fig. 4E), indicating high accuracy and reliability in detecting vascular lesions. Furthermore, spatial agreement of lesions with manual annotations in validation images revealed IoU values of 0.73 (95% CI, 0.71–0.75) and 0.76 (95% CI, 0.72–0.79), respectively, at individual lesion and image levels (Figs. 4F, 4G). The AI method also demonstrated strong agreement in lesion area measurements, as indicated by ICC (0.976) (Figs. 5A, 5B), Bland–Altman analysis (Figs. 5C, 5D), and PE metrics (Figs. 5E, 5F).

Notably, these performance metrics on the validation dataset closely matched those observed during training (Figs. 1^2^–3). Furthermore, the performance of the AI model was comparable to, and in some cases exceeded, the interrater agreement among three independent human operators in all of these metrics (Supplementary Figs. S5–S8, Supplementary Table S1, Table). This result underscores the capability of the AI model to achieve expert-level accuracy, making it a potentially valuable tool in preclinical studies of retinal neovascular diseases.

### AI Performance on Measuring Vascular Leakage

**Figure 6.**
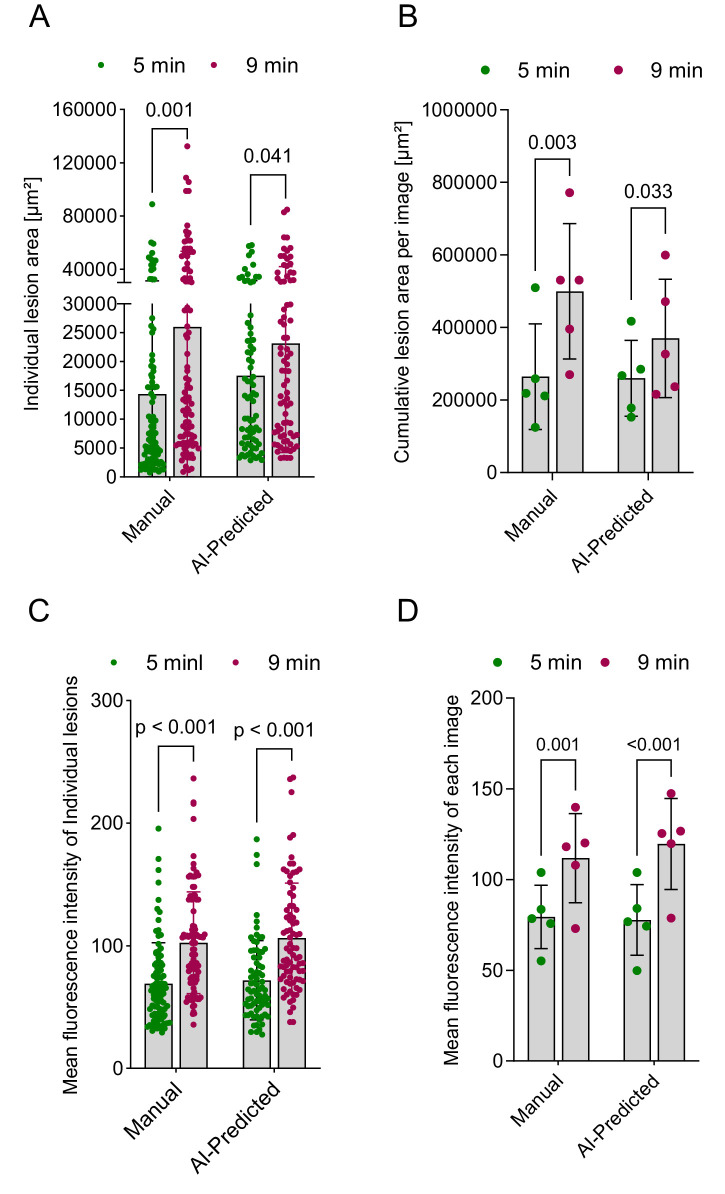
Evaluation of the AI method performance in measuring vascular leakage. (**A**) Manual and AI-assisted assessment of vascular leakage as measured by expansion of lesion area. Areas of individual lesions are plotted. *P* < 0.05 (two-tailed unpaired *t*-test) indicates significance. (**B**) Manual and AI-assisted assessment of vascular leakage as measured by expansion of lesion area. Cumulative lesion areas of individual images are plotted. *P* < 0.05 (two-tailed paired *t*-test) indicates significance. (**C**) Manual and AI-assisted assessment of vascular leakage by measuring the mean fluorescence intensity of individual lesions. The fluorescence intensities of individual lesions are plotted. *P* < 0.05 (two-tailed unpaired *t*-test) indicates significance. (**D**) Manual and AI-assisted assessment of vascular leakage. Mean fluorescence intensities across all lesions within each image are plotted. *P* < 0.05 (two-tailed paired *t*-test) indicates significance.

We next evaluated the performance of the AI model in a real-world scenario by quantifying vascular leakage in *Vldlr^−/−^* mice. FFA images were captured at 5 and 9 minutes post-fluorescein administration, and lesion areas were measured and compared. The AI model successfully quantified that lesion sizes in the 9-minute cohort were significantly larger than those in the 5-minute cohort both at the level of individual lesion and total lesion area per image (Figs. 6A, 6B), consistent with results obtained through manual segmentation of the same image set. Because our AI method accurately identified and delineated the lesions, we next examined vascular leakage intensity by measuring the MFI for individual lesions, as well as the average MFI across all lesions within each image. As expected, the AI method performed comparably to the manual method in quantifying leakage at both the individual lesion level (Fig. 6C) and the whole-image level (Fig. 6D).

### Evaluation of AI Model Performance on Peripheral Lesion Detection and Quantification

**Figure 7.**
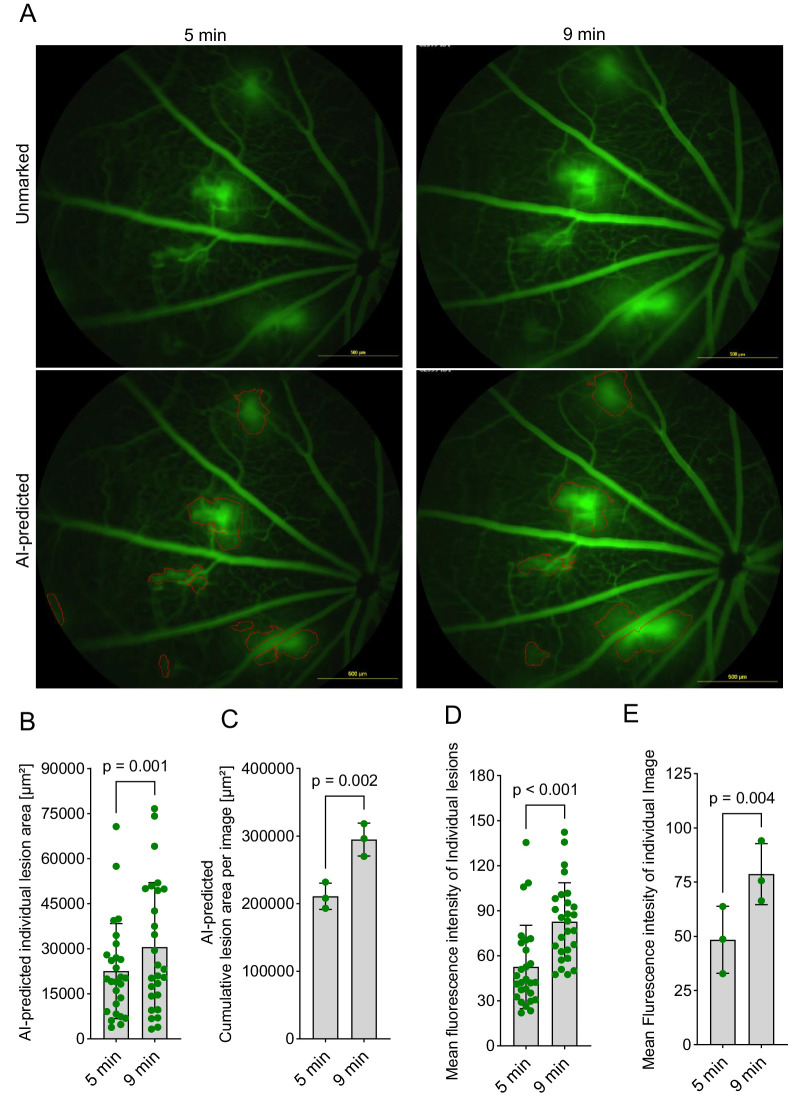
Evaluation of the AI model performance on peripheral lesion detection and quantification. (**A**) Representative FFA images acquired at 5 and 9 minutes, highlighting the ability of the AI model to accurately segment lesions in peripheral regions where the optic nerve head is not centered are presented. (**B**) AI-assisted assessment of vascular leakage in peripheral lesions as measured by expansion of lesion area. Areas of individual lesions are plotted. *P* < 0.05 (two-tailed unpaired *t*-test) indicates significance. (**C**) AI-assisted assessment of vascular leakage in peripheral lesions as measured by expansion of lesion area. Cumulative lesion areas of individual images are plotted. *P* < 0.05 (two-tailed paired *t*-test) indicates significance. (**D**) AI-assisted assessment of vascular leakage in peripheral lesions, by measuring the mean fluorescence intensity of individual lesions. The fluorescence intensities of individual lesions are plotted. *P* < 0.05 (two-tailed unpaired *t*-test) indicates significance. (**E**) AI-assisted assessment of vascular leakage in peripheral lesions, by measuring the mean fluorescence intensity across all lesions within each image are plotted. *P* < 0.05 (two-tailed paired *t*-test) indicates significance.

All of the images used for AI training and validation were acquired with the optic nerve head centered in the field of view. To assess the versatility of the model, we tested its ability to quantify vascular lesions in peripheral regions. Despite not being specifically trained on such images, the AI model effectively detected and delineated the peripheral lesion boundaries (Fig. 7A). Additionally, our AI method also demonstrated temporal expansion of the lesion area (Figs. 7B, 7C) and leakage intensity (Figs. 7D, 7E). These findings demonstrate the versatility and robustness of our AI model in accurately quantifying vascular leakage across varying conditions, including differences in imaging time points and lesion locations. The ability of the model to generalize to peripheral lesions, despite no training data from non-central regions, highlights its potential for broader application in diverse scenarios.

## Discussion

Our study demonstrates the effectiveness of an AI-based analysis pipeline for quantifying vascular lesions in FFA images of mouse models. Our findings highlight the critical advantages of this approach over traditional manual methods, which are time consuming, susceptible to interobserver variability, and difficult to scale for large datasets. Compared to the methodology by Comin et al.,^1^ which employed log-Gabor quadrature filters and morphological operations for vascular leakage detection, our study introduces an AI-based approach trained on manually segmented FFA images. Although both methods aim for automated quantification of vascular leakage, they differ significantly in scalability, robustness, and manual intervention requirements.

The method by Comin et al.^1^ relies on intensity thresholding, making it sensitive to background noise and imaging artifacts, which can compromise accuracy under diverse imaging conditions, and it necessitates frequent adjustment of thresholding parameters. In contrast, our AI model leverages learned feature recognition to adapt to complex variations in lesion morphology and fluorescence intensity, reducing susceptibility to noise and artifacts while enabling autonomous high-throughput analysis across large datasets. Additionally, although the method of Comin et al.^1^ was validated exclusively on the *Vldlr^−/−^* mouse model, our approach demonstrated broader applicability by being validated on multiple preclinical models, including both the *Vldlr^−/−^* and laser-induced CNV models. A key advantage of our pretrained AI model is its ability to be directly applied to analyze new FFA images without requiring further training or large annotated datasets. This capability significantly reduces the time and effort required for analysis, allowing researchers to quickly and accurately analyze their images without needing coding expertise.

To facilitate widespread adoption of our AI method, we have provided the Segment Objects AI (.oai) file and General Analysis 3 (.ga3) file, along with step-by-step user instructions (Supplementary Methods). The Segment Objects AI (.oai) file contains a pretrained AI model optimized for identifying mouse FFA vascular lesions, and the GA3 file enables automated lesion counting, area measurement, intensity analysis, and data tabulation. Researchers can easily import these files into NIS-Elements and analyze their images in a plug-and-play fashion, making advanced image analysis accessible to a wider research community.

The AI model achieved high levels of agreement with manual annotations across key measurements such as lesion count, spatial overlap (IoU), area, and leakage intensity. Notably, the performance of the model often surpassed the interobserver variability among human annotators, indicating a high degree of reliability and reproducibility. The model also demonstrated robust generalization capabilities, maintaining consistent performance on both training and unseen validation datasets. Importantly, the AI tool effectively captured the expected temporal changes in vascular leakage in *Vldlr^−/−^* mice between 5- and 9-minute FFA captures, suggesting its suitability for real-world experimental conditions. Importantly, its performance remained consistent across both mouse models (laser CNV and *Vldlr^−/−^*), highlighting its adaptability and robustness in diverse pathological contexts.

For a small subset of images, lesion count and area varied significantly between the AI and manual methods. These discrepancies primarily arose from inconsistencies in user-defined lesion boundaries, particularly when two or more closely located lesions had touching or partially overlapping boundaries (Supplementary Fig. S2). However, whereas individual lesion areas could show considerable variation (Fig. 3C), the total lesion area per image (Fig. 2E) remained largely consistent between the AI and manual methods, suggesting that the observed differences were primarily due to the merging of adjacent lesions rather than systematic measurement errors.

In defining the “true” leakage area in FFA images, a challenge arises due to the diffuse and gradual nature of leakage margins. To address this, the ground truth was established based on manual annotations, where trained graders delineated the leakage boundaries by considering intensity thresholds relative to the background and anatomical context. Given the inherent ambiguity in identifying leakage margins, the AI model was trained on a dataset that accounted for human-annotated variability. Specifically, seven images were manually segmented by three independent operators, yielding 21 unique training images, as detailed in the Methods section. Furthermore, the performance of the model was evaluated by comparing its outputs to both individual grader annotations and interobserver variability, demonstrating strong alignment with human assessments. This approach underscores the robustness of the AI model in handling the challenges associated with leakage margin delineation.

With the ability to accurately analyze hundreds of images within minutes, our model eliminates the need for time-consuming manual annotation. This enables large-scale studies that have previously been impractical. In conclusion, by streamlining FFA lesion analysis to be faster, more reliable, and easily scalable while maintaining accuracy and precision, this approach holds the potential to greatly improve research efficiency in studies of vascular pathophysiology, particularly in preclinical models of neovascular conditions such as AMD.

### Limitations and Future Directions

Our AI model was trained exclusively on images from the MICRON IV fundus imaging system. Consequently, its performance on images from other platforms remains uncertain due to potential domain shift. Variations in image characteristics (resolution, contrast, illumination, field of view) and preprocessing requirements across different cameras could impact accuracy. However, the successful quantification by the model of peripheral vascular lesions in FFA images, a task outside its training domain, suggests a degree of robust feature recognition. This indicates potential for generalization to other imaging platforms, provided that the essential vascular lesion features are preserved. Future studies incorporating diverse imaging systems, along with potential fine-tuning or domain adaptation, could help enhance the generalizability of our model across diverse imaging platforms, further strengthening its applicability to preclinical and translational research.

## Supplementary Material

Supplement 1

Supplement 2

Supplement 3

Supplement 4
