# Supplementary material for: AI Quantification of Vascular Lesions in Mouse Fundus Fluorescein Angiography
Source: Transl Vis Sci Technol. 2025 Jun 2;14(6):4. doi: 10.1167/tvst.14.6.4 (PMC12136100; doi:10.1167/tvst.14.6.4)
Supplement: Supplement 1 [file tvst-14-6-4_s001.pdf]

Supplementary Material

AI Quantification of Vascular Lesions in Mouse Fundus Fluorescein Angiography

Vinodhini Jayananthan[1], Tyler Heisler Taylor[1], David Henry Greentree[1], Bryce Collison[2], Nagaraj Kerur[1, 3]*

1. Department of Ophthalmology and Visual Sciences, College of Medicine, The Ohio State University Wexner Medical Center, Columbus, OH, USA

2. Department of Neuroscience, College of Medicine, The Ohio State University Wexner Medical Center, Columbus, OH, USA

3. Department of Microbial Infection and Immunity, College of Medicine, The Ohio State University Wexner Medical Center, Columbus, OH, USA

* Correspondence and requests for materials should be addressed to N.K. (nagaraj.kerur@osumc.edu).

Supplementary Figure 1

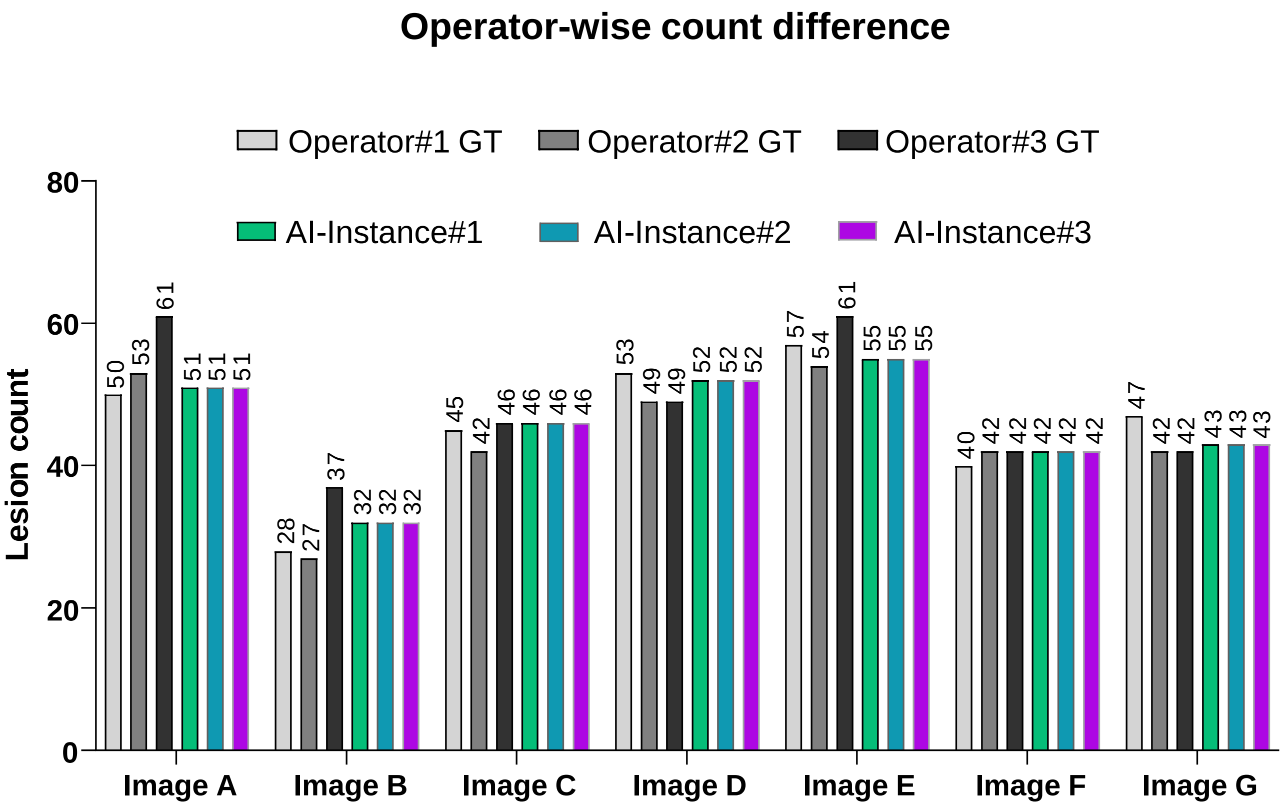

Supplementary Figure 2

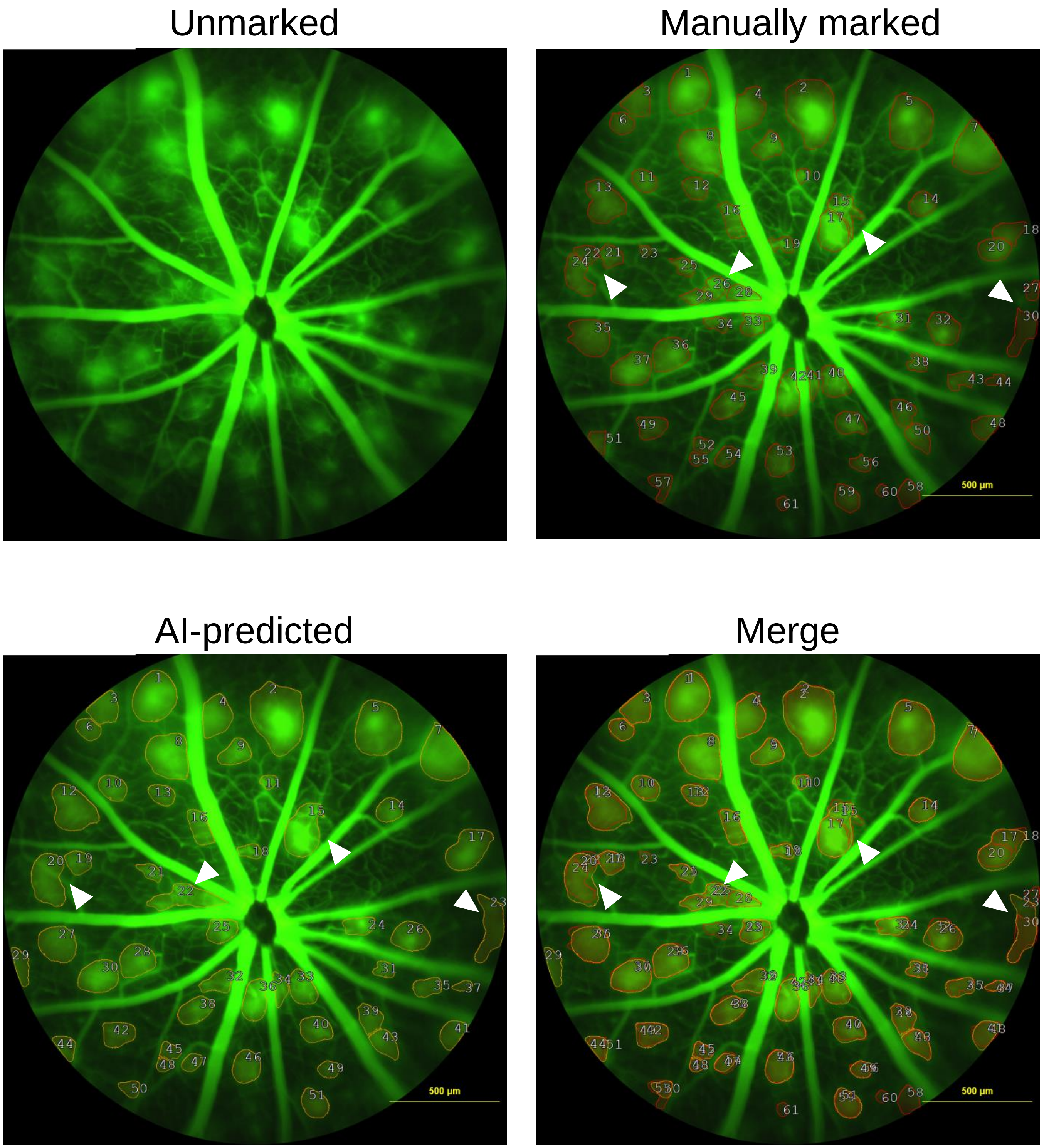

Supplementary Figure 3

Manual

AI-Predicted

PE= -0.03

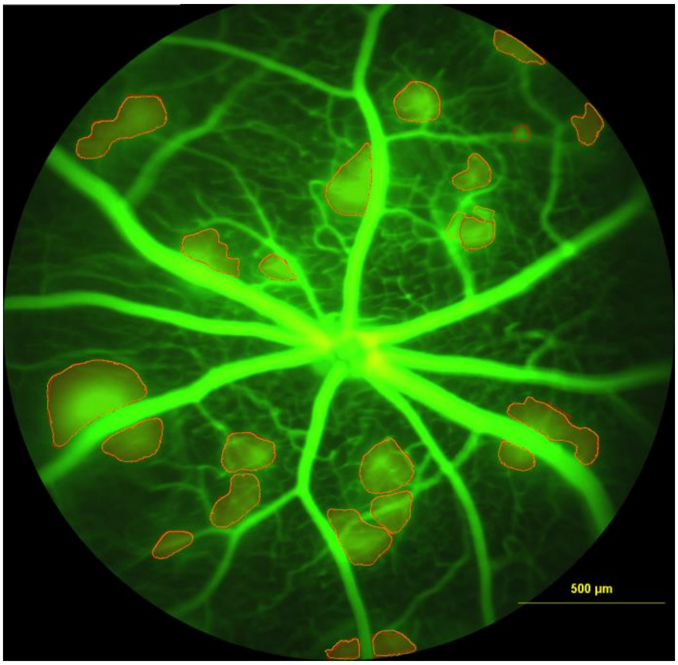

PE= -1.94

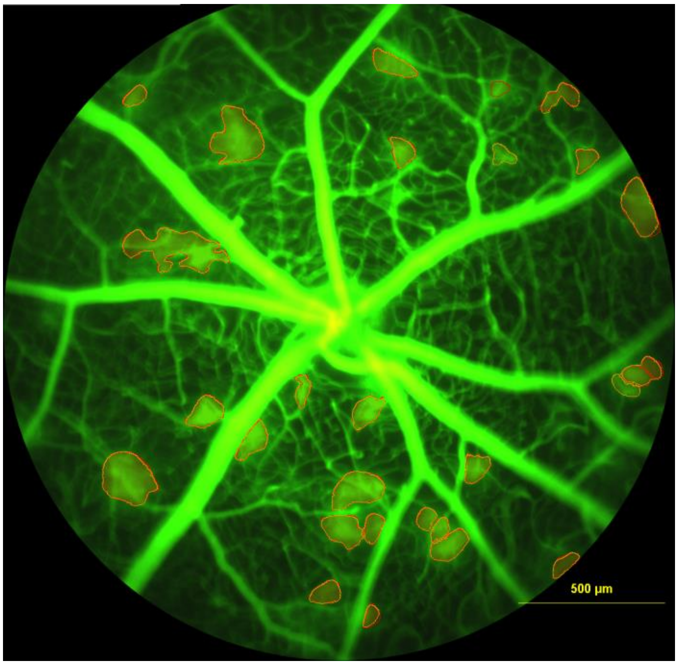

Manual

AI-Predicted

PE= -0.35

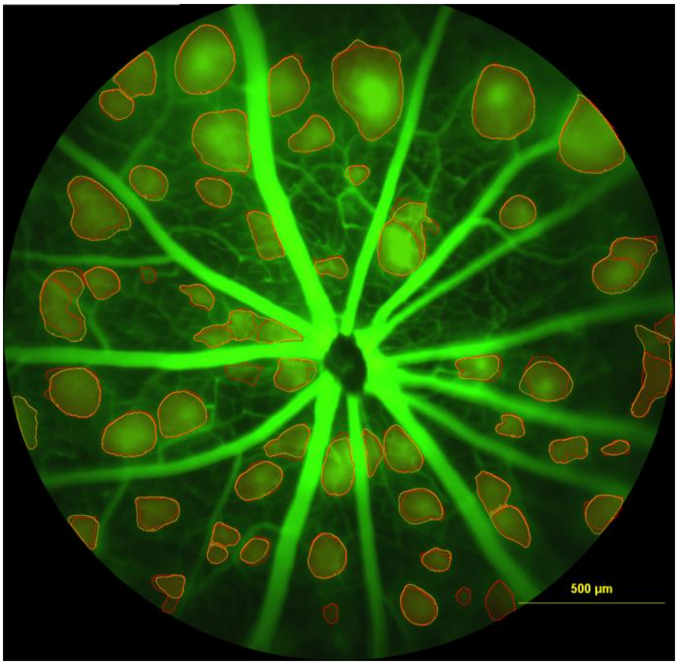

PE= 7.9

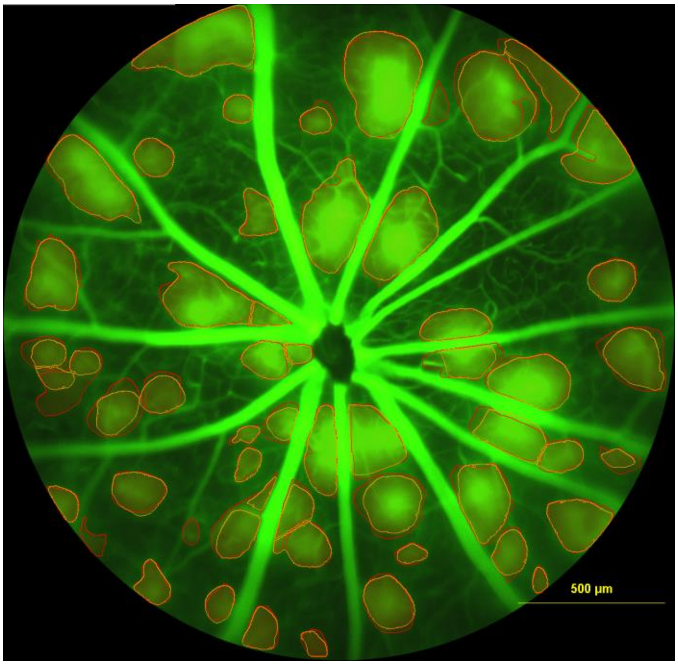

Supplementary Figure 4

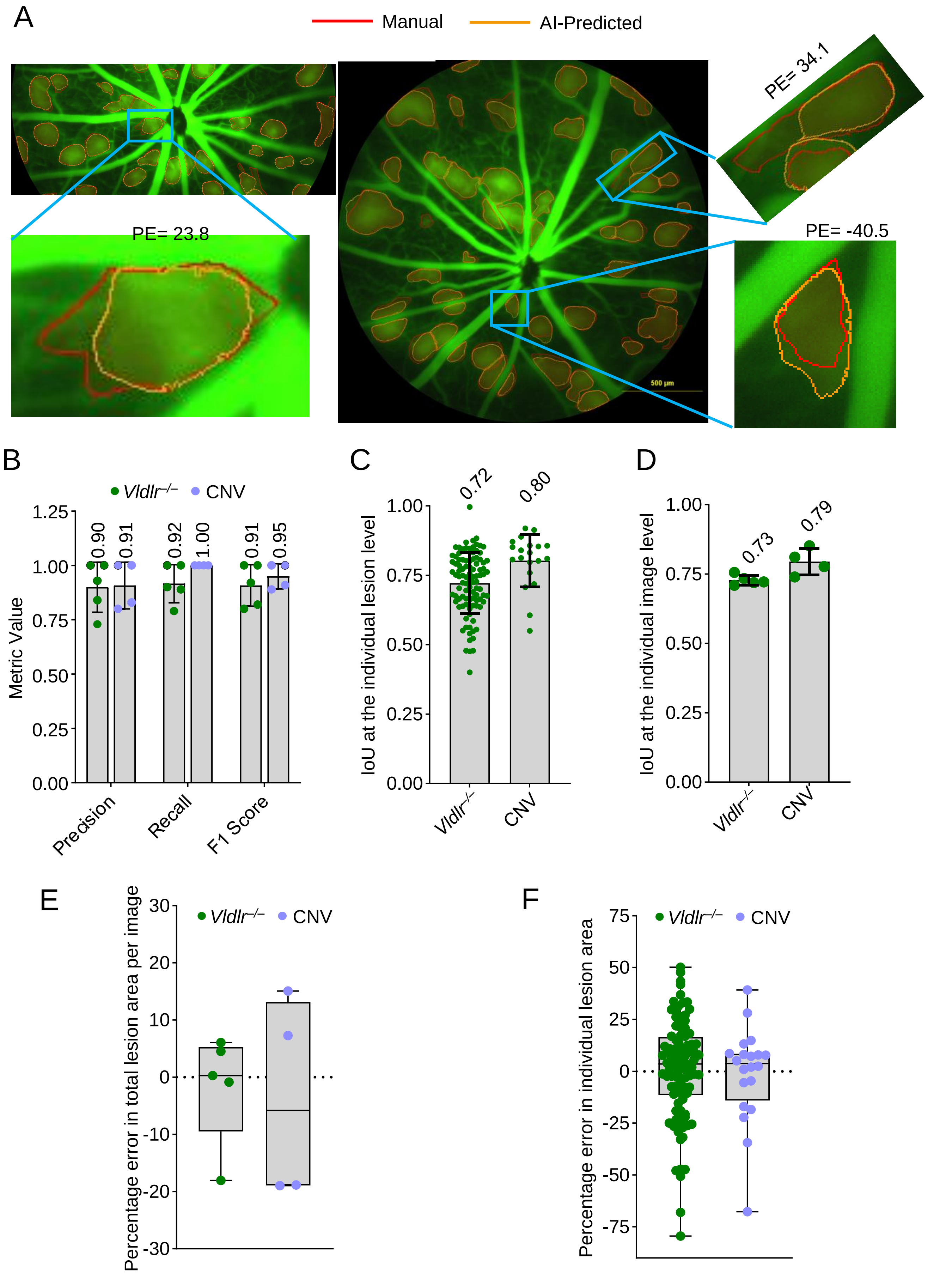

Supplementary Figure 5

A

Correlation analysis lesion count per image between two users with intraclass correlation coefficient (ICC)

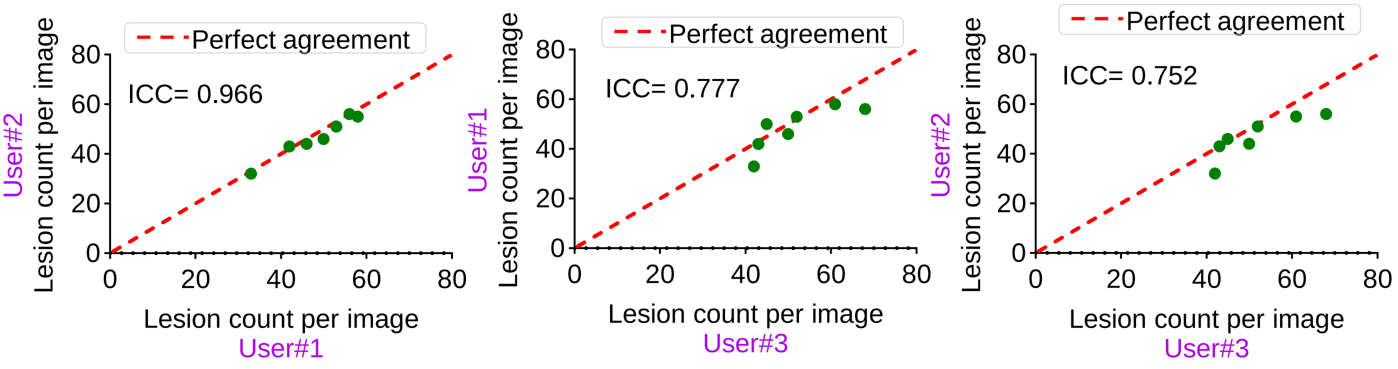

B

Comparison of lesion count per image between two users using a Bland-Altman plot

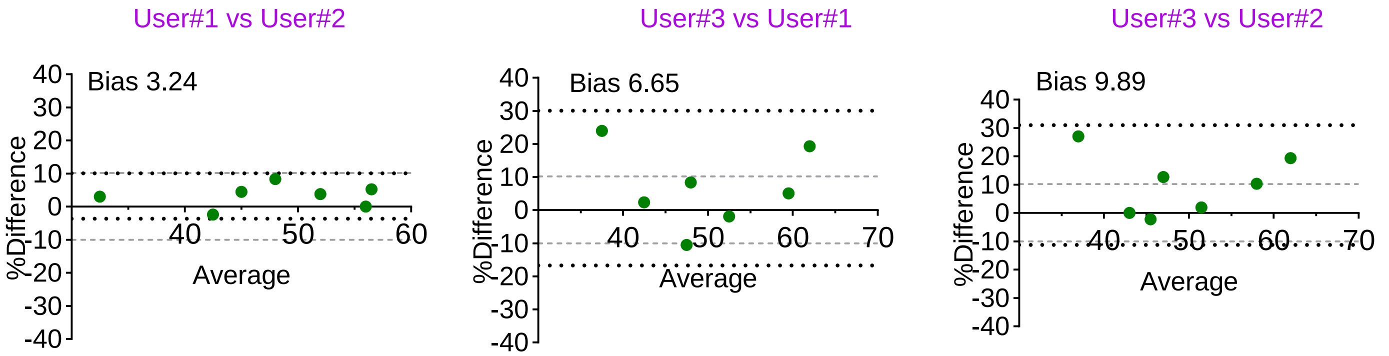

C

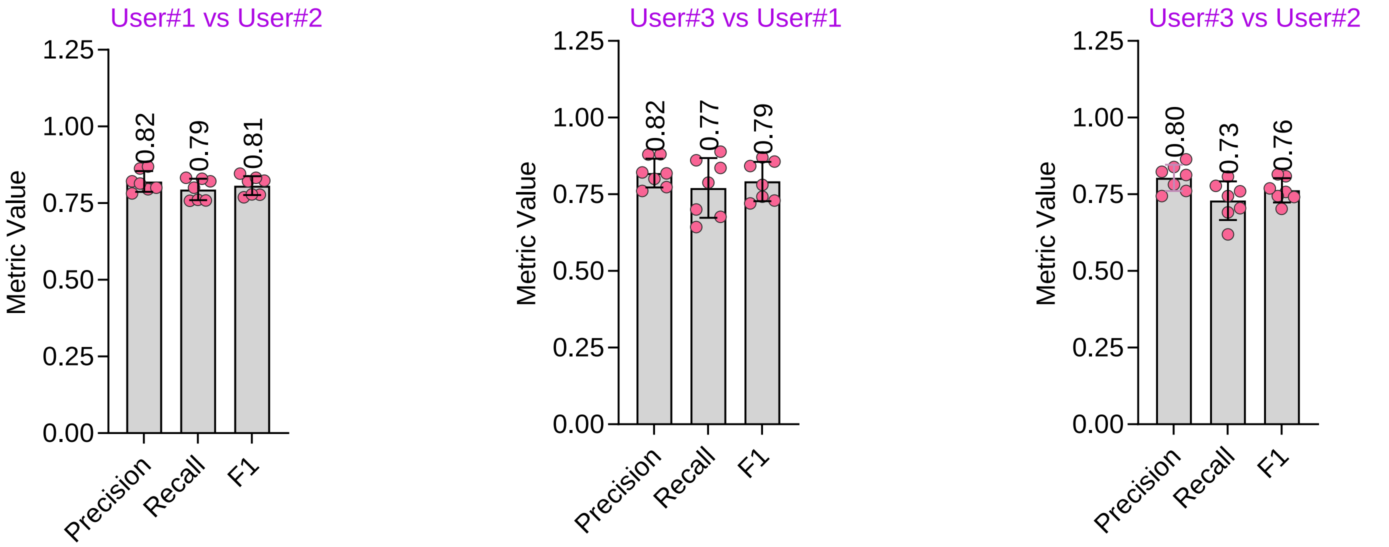

Supplementary Figure 6

A

Correlation analysis of individual lesion Area measurements between two users with intraclass correlation coefficient (ICC)

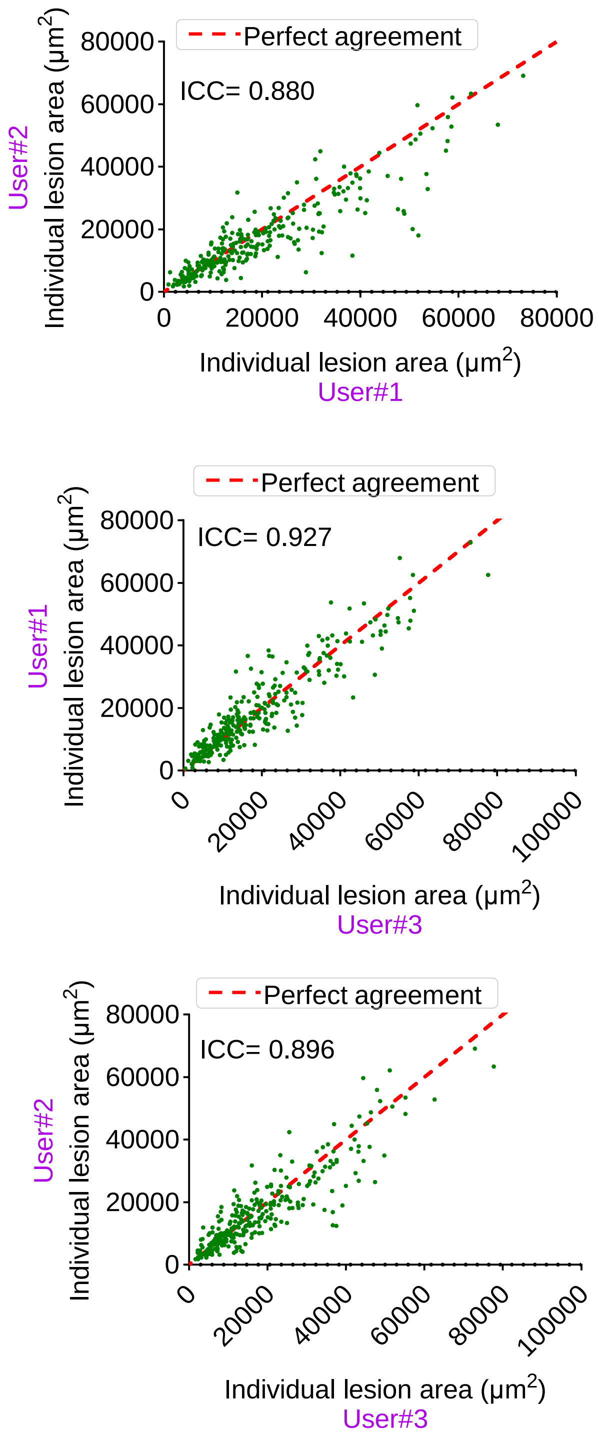

B

Comparison of individual lesion area measurements between two users using a Bland-Altman plot

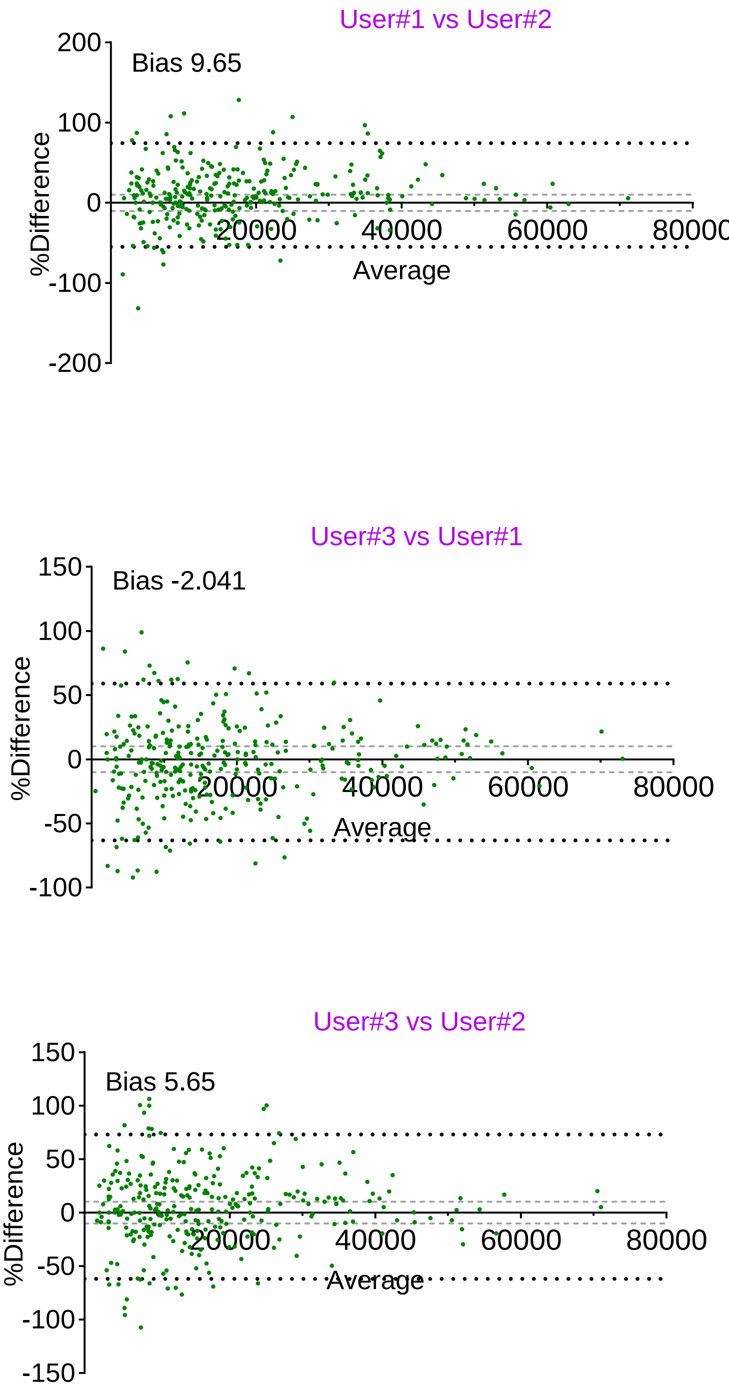

Supplementary Figure 7

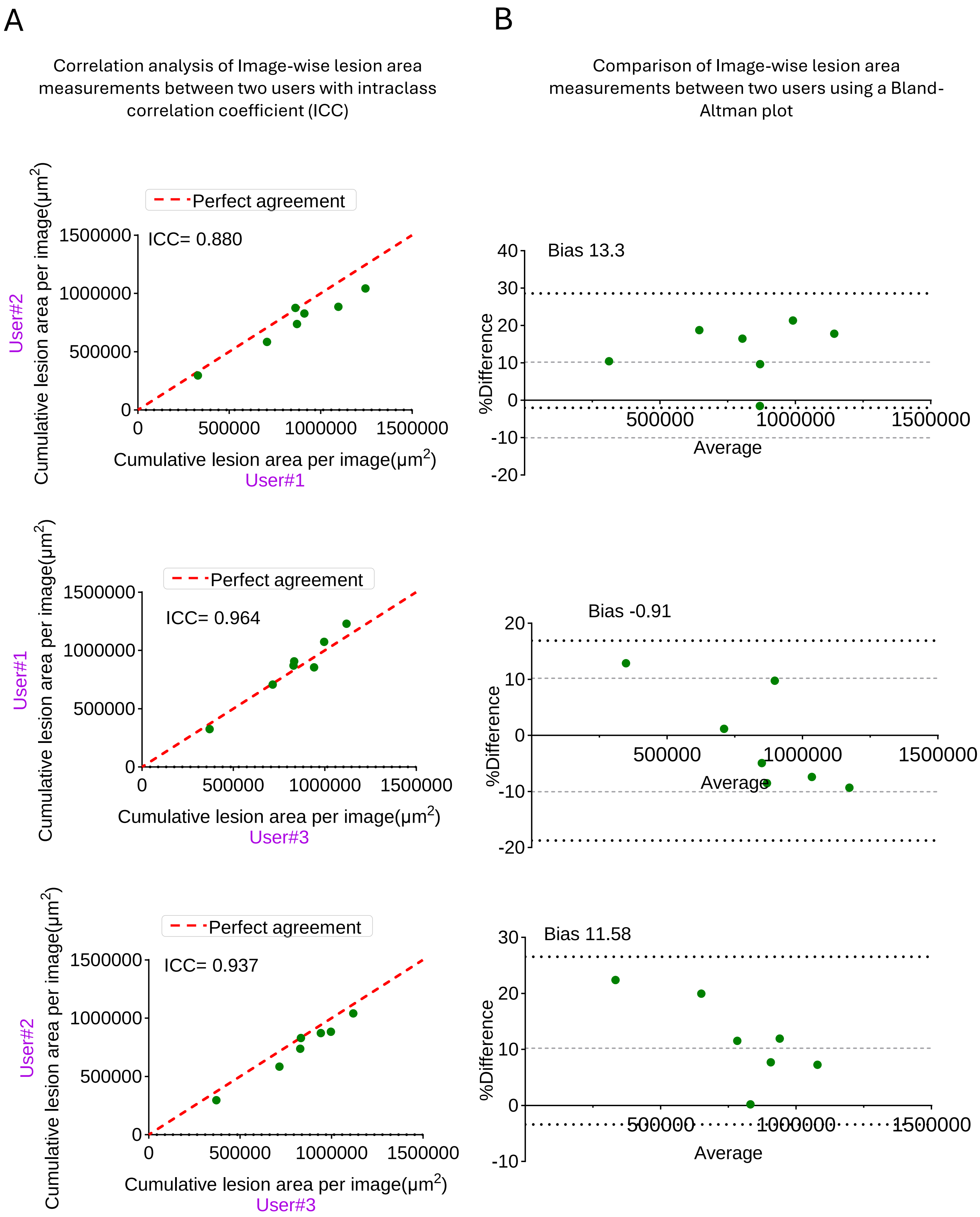

Supplementary Figure 8

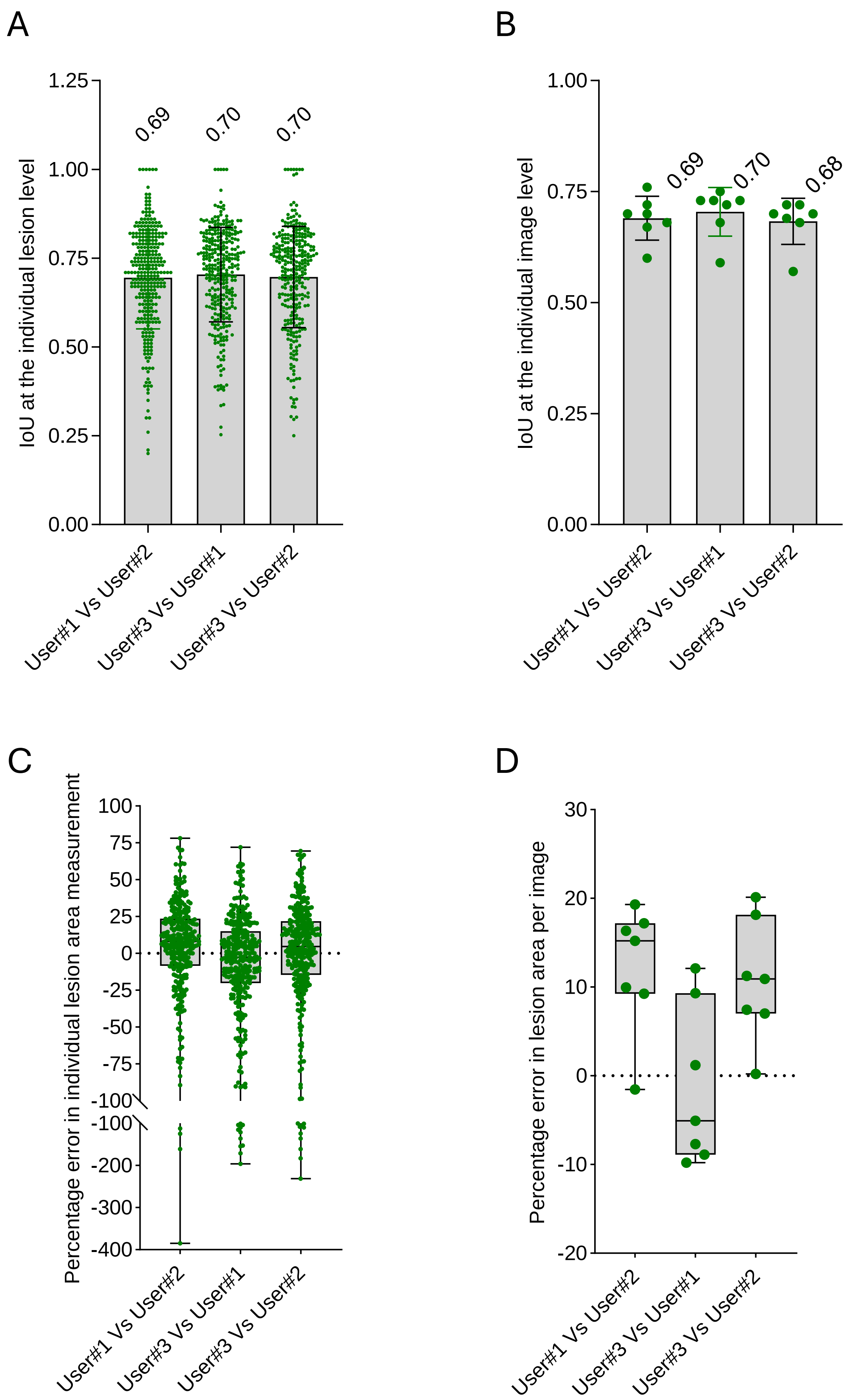

Ste-by-step guide for Implementation of AI method in NIS Elements software

These instructions will guide the user through importing and configuring the necessary files to utilize our AI model for automated analysis of mouse FFA vascular lesions. NIS Elements software with the AI suite (version 6.02.03 is recommended) is required (see details below). These instructions are designed for batch processing of multiple images within the software.

- **Step 1**-through **Step 3** describe image pre-processing.
- **Step 4** provides instructions for importing and preparing the Segment Objects AI (.oai) file and General Analysis 3 (.ga3) file into the user's NIS-Elements software. The Segment Objects AI (.oai) file includes a pre-trained AI model designed to identify and delineate mouse FFA vascular lesions, while the GA3 file contains a recipe for lesion counting, area measurement, intensity analysis, and data tabulation.
- **Step 5** describes instructions on image analysis using the AI method

Important considerations:

- **Software Version:** Our AI method was generated using NIS-Elements version 6.02.03. We strongly recommend using this version or consulting Nikon to ensure compatibility between your version of the software and our .oai and GA3 files.
- **Imaging platform:** The AI model was trained on FFA images acquired on Micron IV. Consequently, its performance on images from other platforms remains uncertain. Variations in image characteristics (resolution, contrast, illumination, field of view) and preprocessing requirements across different cameras could impact accuracy.

Step 1: Convert files to ND2 format

File>Import/Export>Convert files> Select folder of images to be converted to>select ".nd2" from the Output File Format from dropdown menu> Select output directory>click the "Convert" button

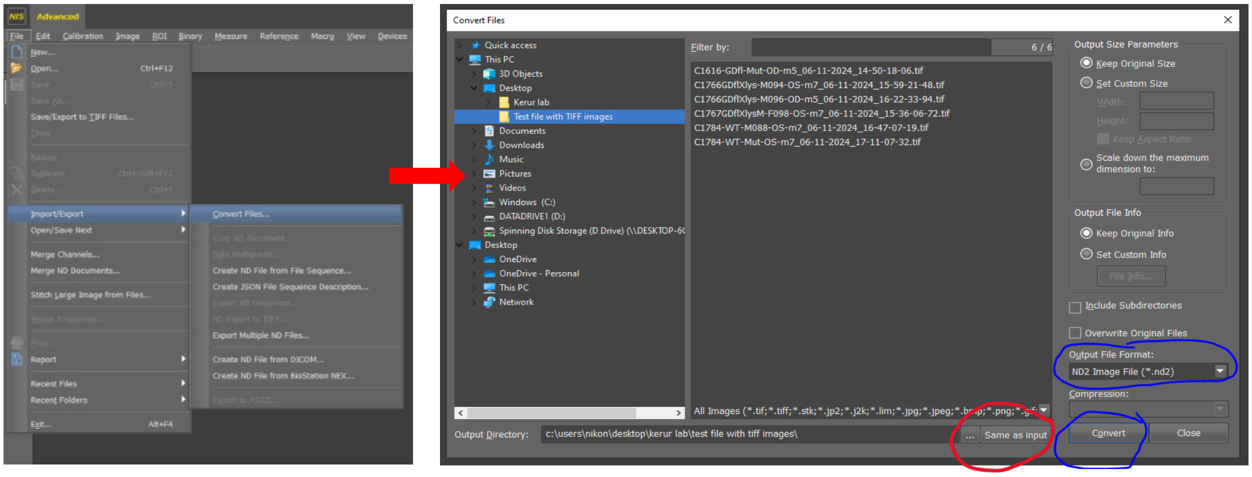

Step 2: Convert the ND2 files into multichannel images using Macro

Macro>Run on files in Folder>select Command/Macro> select Command list >chose “ConvertToMCH()” from the list> select folder of ND2 files you want convert to multichannel images> see image below on the right for other options> click Run

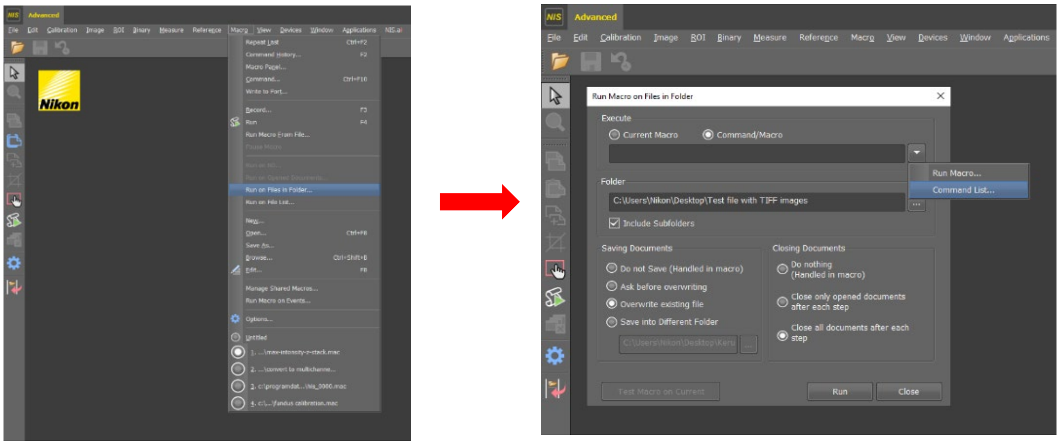

Step 3: Calibrate Multi channel ND2 files from step 2.

In this step, the images are calibrated using pixel size. We have used 2.1µm/pixel for calibrating FFA images acquired using Micron IV imaging system. This information will vary from one imaging platform to another. As discussed in the manuscript, we have not tested this AI method on images acquired from non-Micron IV systems.

1. Macro>Run on files in Folder>select Command/Macro> select Run Macro> choose “fundus calibration.mac” from a saved location*> select folder of ND2 files resulting from Step 2 processing> see image below on the right for other options>Run

   *We have provided fundus calibration macro file “fundus calibration.mac”. download it and use it at this step for images acquired on Micron IV system

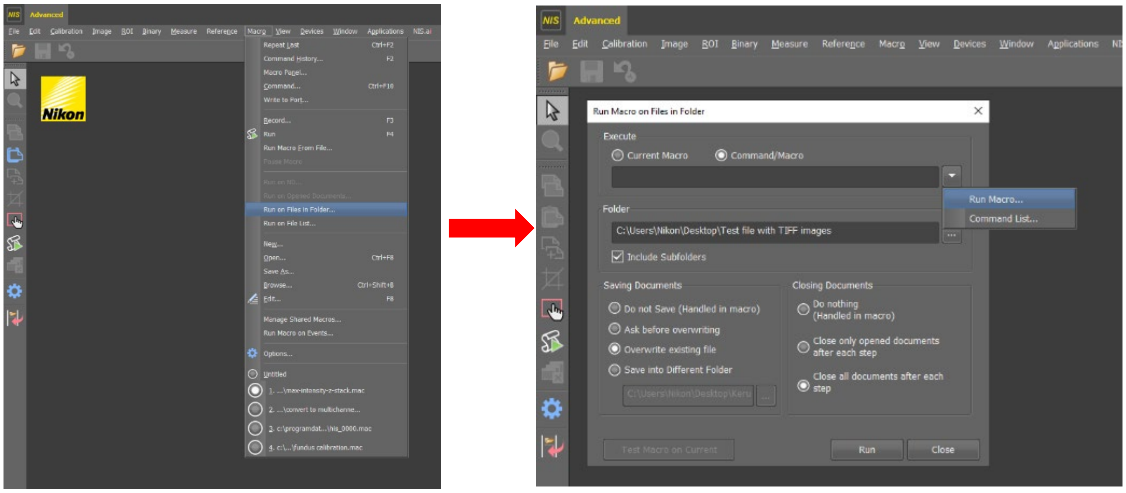

2. These files are now ready to be analyzed by the AI method. Save these files in a folder and note the location for use in the next step analysis.

Step 4: Importing necessary files into NIS Elements software (this needs to be done only once)

a) **Download the following files and save on your computer, taking note of their location**
   a. Segment Objects AI (.oai file): This is pretrained AI model
   b. General Analysis 3(.ga3 file): This analysis recipe

b) **Follow the steps below to import GA3 file and use the downloaded Segment Objects AI (.oai file)**

1. Launch the NIS Elements software
2. Right click>Analysis controls>Analysis explorer
   a. A new "Analysis Explorer" panel will open
   b. Click Create new
   c. Select "Import Analysis From File"
   d. Navigate to the location where you saved the GA3 file and select it (you have to do this only once at the very first time)

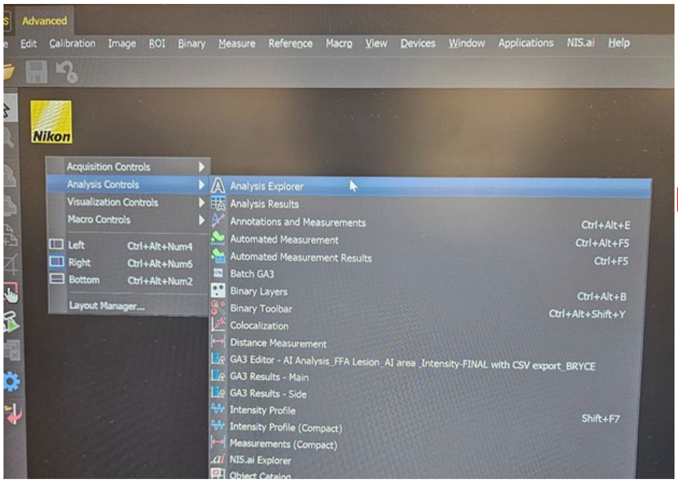

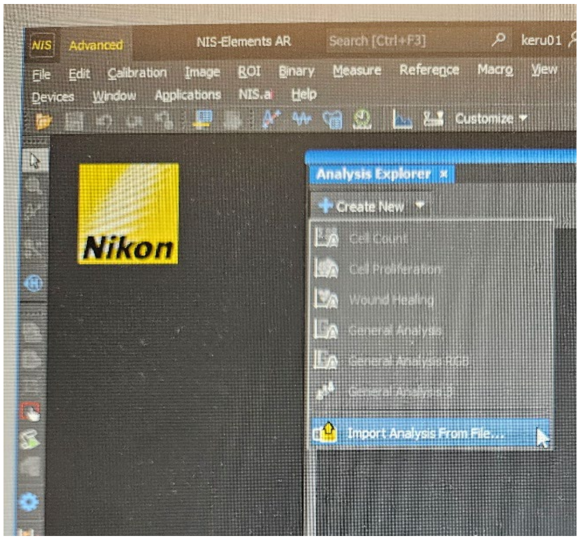

3. At this stage you should see the imported GA3 file under Analysis explorer

4. Highlight imported GA3 file and click edit to open it in GA3 editor

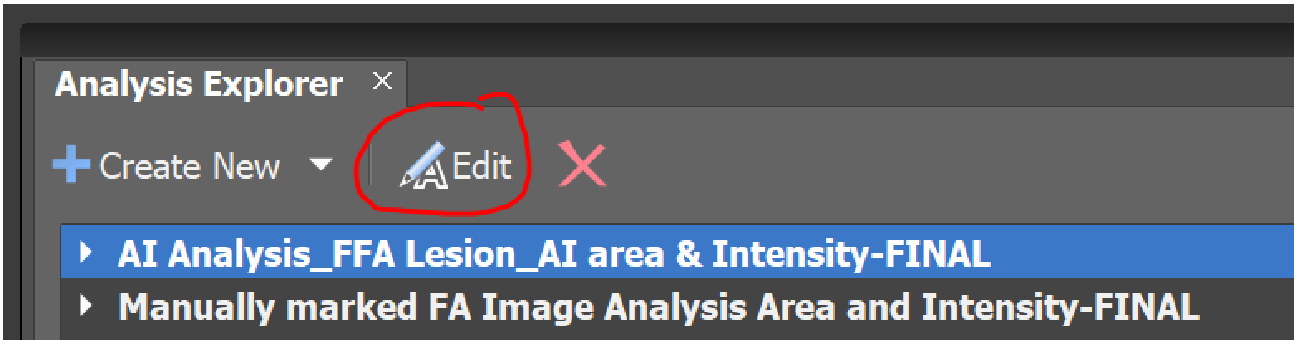

5. In the GA3 Editor panel, Click on three dots next to SegmentObjects_ai.
   a. New SegmentObjects_ai selection panel will open
   b. Click “browse” and select the provided Segment Objects AI (.oai) file

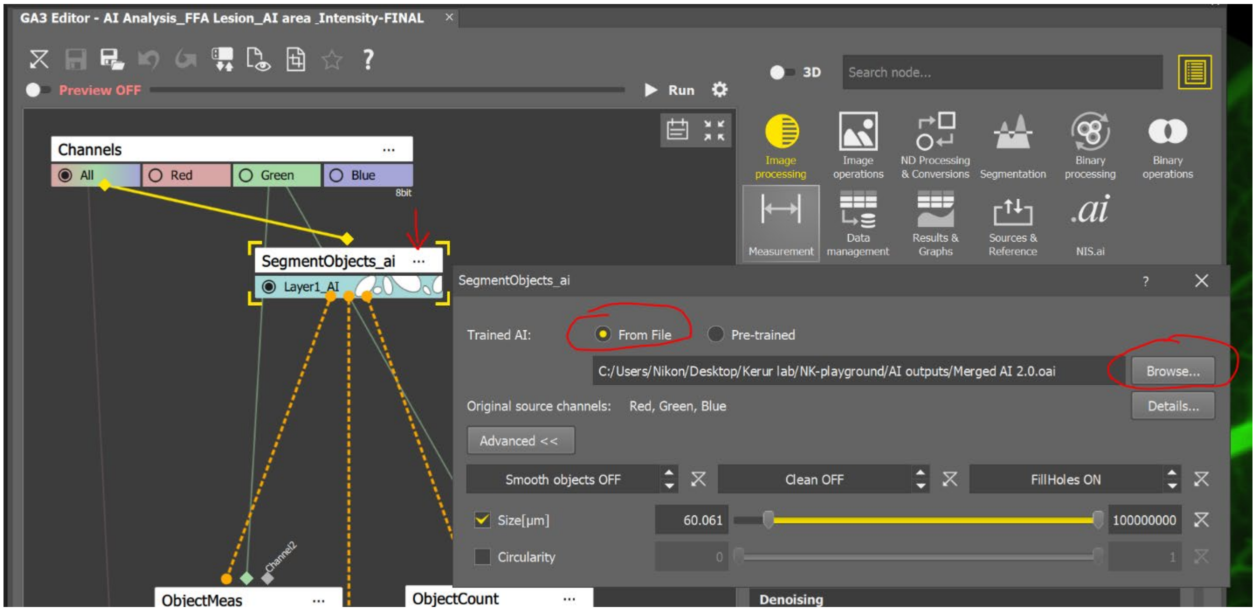

6. Click “Save”

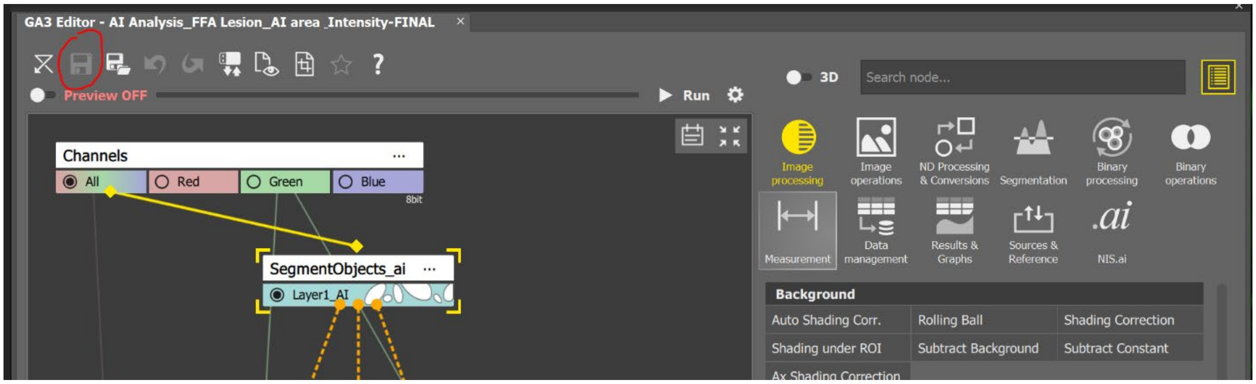

The GA3 file is now configured with a pretrained AI model (.oai file) and is ready for image analysis. You will use this GA3 file from this point forward to analyze your images.

STEP 5: Analyze FFA images processed by step 1 through 3 using the AI method.

- **This method is meant analyze all ND2 files saved within a desired folder**

1. Open NIS element> Right click>Analysis controls>Batch GA3>Add folder >select the GA3 file prepared in STEP 4 above.
   a. Next, you will be prompted to select the folder containing the images to be analyzed (these are the files obtained after completing steps 1 through 3)

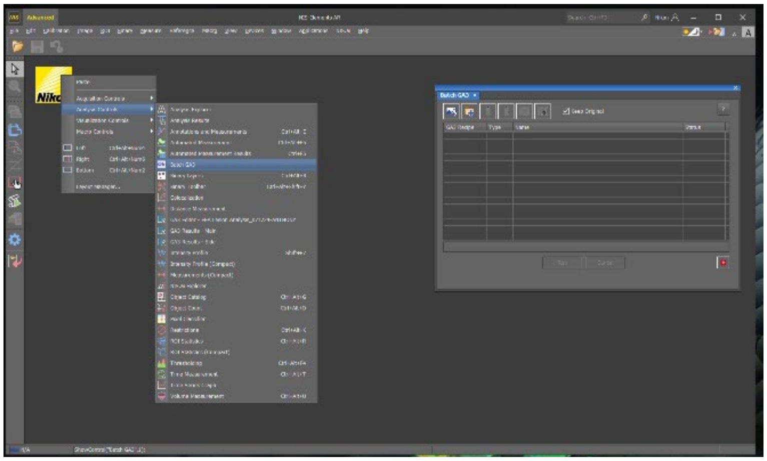

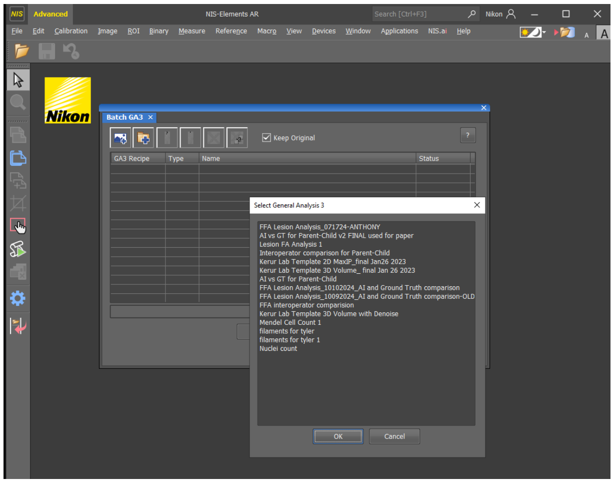

   b. This should provide you with a table of data in an "Analysis Results" window inside NIS Elements. You should also have a .csv file containing data for each image in the folder you specified in the previous step

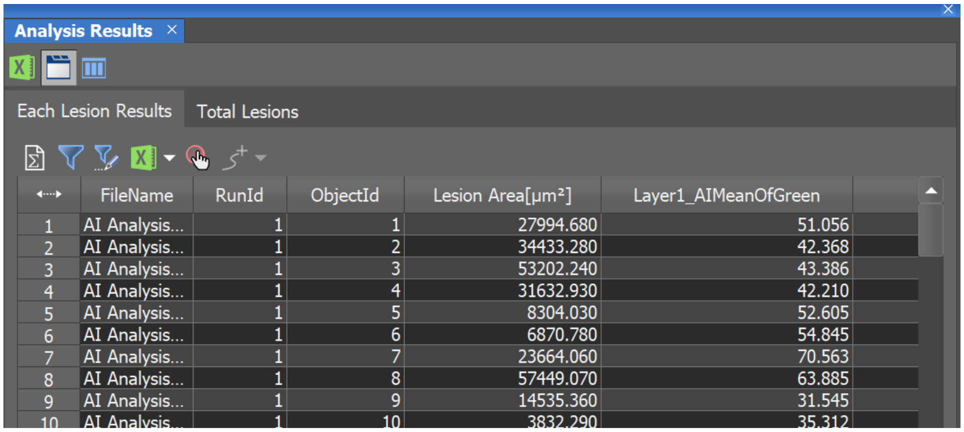

	FileName	RunId	ObjectId	Lesion Area[µm²]	Layer1_AIMeanOfGreen
1	AI Analysis...	1	1	27994.680	51.056
2	AI Analysis...	1	2	34433.280	42.368
3	AI Analysis...	1	3	53202.240	43.386
4	AI Analysis...	1	4	31632.930	42.210
5	AI Analysis...	1	5	8304.030	52.605
6	AI Analysis...	1	6	6870.780	54.845
7	AI Analysis...	1	7	23664.060	70.563
8	AI Analysis...	1	8	57449.070	63.885
9	AI Analysis...	1	9	14535.360	31.545
10	AI Analysis...	1	10	3832.290	35.312

   c. The downloaded CVS file will have data for lesion-wise and image-wise lesional area and fluorescence intensity (see example below)

FileName	RunId	TimeLapseIndex	Entity	Object Id	Area-Each Lesion	MeanOfGreen-Each Lesion	FileName	RunId	Lesion Count	Total Lesion Area Per Image	MeanOfGreen Per Image
A-5min-C2399	0	1	Layer1_AI	1	37432.1	52.26	A-5min-C239	1	8	227507.5	59.14
A-5min-C2399	0	1	Layer1_AI	2	93836.0	59.32	A-5min-C239	1	8	227507.5	59.14
A-5min-C2399	0	1	Layer1_AI	3	8661.2	42.80	A-5min-C239	1	8	227507.5	59.14
A-5min-C2399	0	1	Layer1_AI	4	10945.6	48.59	A-5min-C239	1	8	227507.5	59.14
A-5min-C2399	0	1	Layer1_AI	5	13865.0	50.51	A-5min-C239	1	8	227507.5	59.14
A-5min-C2399	0	1	Layer1_AI	6	13172.7	62.87	A-5min-C239	1	8	227507.5	59.14
A-5min-C2399	0	1	Layer1_AI	7	41365.8	79.85	A-5min-C239	1	8	227507.5	59.14
A-5min-C2399	0	1	Layer1_AI	8	8229.1	24.15	A-5min-C239	1	8	227507.5	59.14

Lesion-wise data

Image-wise data
